# Dynamics and heterogeneity of brain damage in multiple sclerosis

**DOI:** 10.1371/journal.pcbi.1005757

**Published:** 2017-10-26

**Authors:** Ekaterina Kotelnikova, Narsis A. Kiani, Elena Abad, Elena H. Martinez-Lapiscina, Magi Andorra, Irati Zubizarreta, Irene Pulido-Valdeolivas, Inna Pertsovskaya, Leonidas G. Alexopoulos, Tomas Olsson, Roland Martin, Friedemann Paul, Jesper Tegnér, Jordi Garcia-Ojalvo, Pablo Villoslada

**Affiliations:** 1 Center for Neuroimmunology, Institut d'Investigacions Biomèdiques August Pi Sunyer (IDIBAPS), University of Barcelona, Barcelona, Spain; 2 Unit of Computational Medicine, Department of Medicine & Science for Life Laboratory, Karolinska Institute, Stockholm, Sweden; 3 Universitat Pompeu Fabra, Barcelona, Spain; 4 National Technical University of Athens, Athens, Greece; 5 Unit of Neuroimmunology, Karolinska Institute, Stockholm, Sweden; 6 Neuroimmunology and MS Research, Neurology Clinic, University Hospital, University Zurich, Zurich, Switzerland; 7 NeuroCure Clinical Research Center, and the Experimental and Clinical Research Center, Charité Universitätsmedizin Berlin and Max Delbrueck Center for Molecular Medicine Berlin, Berlin, Germany; 8 Biological and Environmental Sciences and Engineering Division & Computer, Electrical and Mathematical Sciences and Engineering Division, King Abdullah University of Science and Technology (KAUST), Thuwal, Kingdom of Saudi Arabia; 9 University of California, San Francisco, United States of America; National Institutes of Health, UNITED STATES

## Abstract

Multiple Sclerosis (MS) is an autoimmune disease driving inflammatory and degenerative processes that damage the central nervous system (CNS). However, it is not well understood how these events interact and evolve to evoke such a highly dynamic and heterogeneous disease. We established a hypothesis whereby the variability in the course of MS is driven by the very same pathogenic mechanisms responsible for the disease, the autoimmune attack on the CNS that leads to chronic inflammation, neuroaxonal degeneration and remyelination. We propose that each of these processes acts more or less severely and at different times in each of the clinical subgroups. To test this hypothesis, we developed a mathematical model that was constrained by experimental data (the expanded disability status scale [EDSS] time series) obtained from a retrospective longitudinal cohort of 66 MS patients with a long-term follow-up (up to 20 years). Moreover, we validated this model in a second prospective cohort of 120 MS patients with a three-year follow-up, for which EDSS data and brain volume time series were available. The clinical heterogeneity in the datasets was reduced by grouping the EDSS time series using an unsupervised clustering analysis. We found that by adjusting certain parameters, albeit within their biological range, the mathematical model reproduced the different disease courses, supporting the dynamic CNS damage hypothesis to explain MS heterogeneity. Our analysis suggests that the irreversible axon degeneration produced in the early stages of progressive MS is mainly due to the higher rate of myelinated axon degeneration, coupled to the lower capacity for remyelination. However, and in agreement with recent pathological studies, degeneration of chronically demyelinated axons is not a key feature that distinguishes this phenotype. Moreover, the model reveals that lower rates of axon degeneration and more rapid remyelination make relapsing MS more resilient than the progressive subtype. Therefore, our results support the hypothesis of a common pathogenesis for the different MS subtypes, even in the presence of genetic and environmental heterogeneity. Hence, MS can be considered as a single disease in which specific dynamics can provoke a variety of clinical outcomes in different patient groups. These results have important implications for the design of therapeutic interventions for MS at different stages of the disease.

## Author summary

Multiple Sclerosis (MS) is an autoimmune disease in which inflammatory and degenerative processes damage the brain. We tested the hypothesis that the variability in disease progression and the clinical heterogeneity observed in MS is driven by a single mechanism, namely the autoimmune attack on the CNS that provokes this chronic inflammation and degeneration. As such, it is the difference in the intensity of these processes at distinct times that is responsible for establishing each of the disease subtypes defined to date. Mathematical models of brain damage and disease course were generated that were fitted to clinical data. We found that the phenotypes of the different MS subtypes were reproduced by the model, supporting the concept of a common pathogenesis and thus, that of a single disease in which specific dynamics can produce a variety of clinical outcomes in different groups of patients. These results are likely to be helpful when designing new therapies for this disease.

## Introduction

Multiple Sclerosis (MS) is an autoimmune disease with a complex pathogenesis that is driven by inflammation and axon degeneration [1]. However, the clinical phenotype of MS is very heterogeneous and the course of the disease is difficult to predict. Neither the frequency of relapses (disease activity) nor the accumulated disability [2] represent an accurate predictor of disease outcome. Relapses in MS have been modeled statistically to a negative binomial distribution [3]. Moreover, relapses have been mathematically modeled from a mechanistic point of view that focuses on the negative feedback between pro- and anti-inflammatory responses [4], and as a probabilistic response to self-antigen presentation [5]. However, how damage to the central nervous system (CNS) advances and how clinical disability accumulates over decades, defining the clinical phenotype of the disease and its prognosis, are issues that are still poorly understood [6]. Several hypotheses have been proposed to explain the heterogeneity and different courses of the disease. These range from considering MS as a single disease to defining it as a disease with two distinct physiological stages (inflammatory and neurodegenerative), or even as different diseases with relapse-remitting MS (RRMS) being defined as an autoimmune disease (the outside-in hypothesis) and primary-progressive MS (PPMS) a primary neurodegenerative disease (the inside-out hypothesis) [7–10]. Alternatively, each of the four pathological patterns described in acute plaques may reflect distinct pathogenic mechanisms [11].

**Fig 1:**
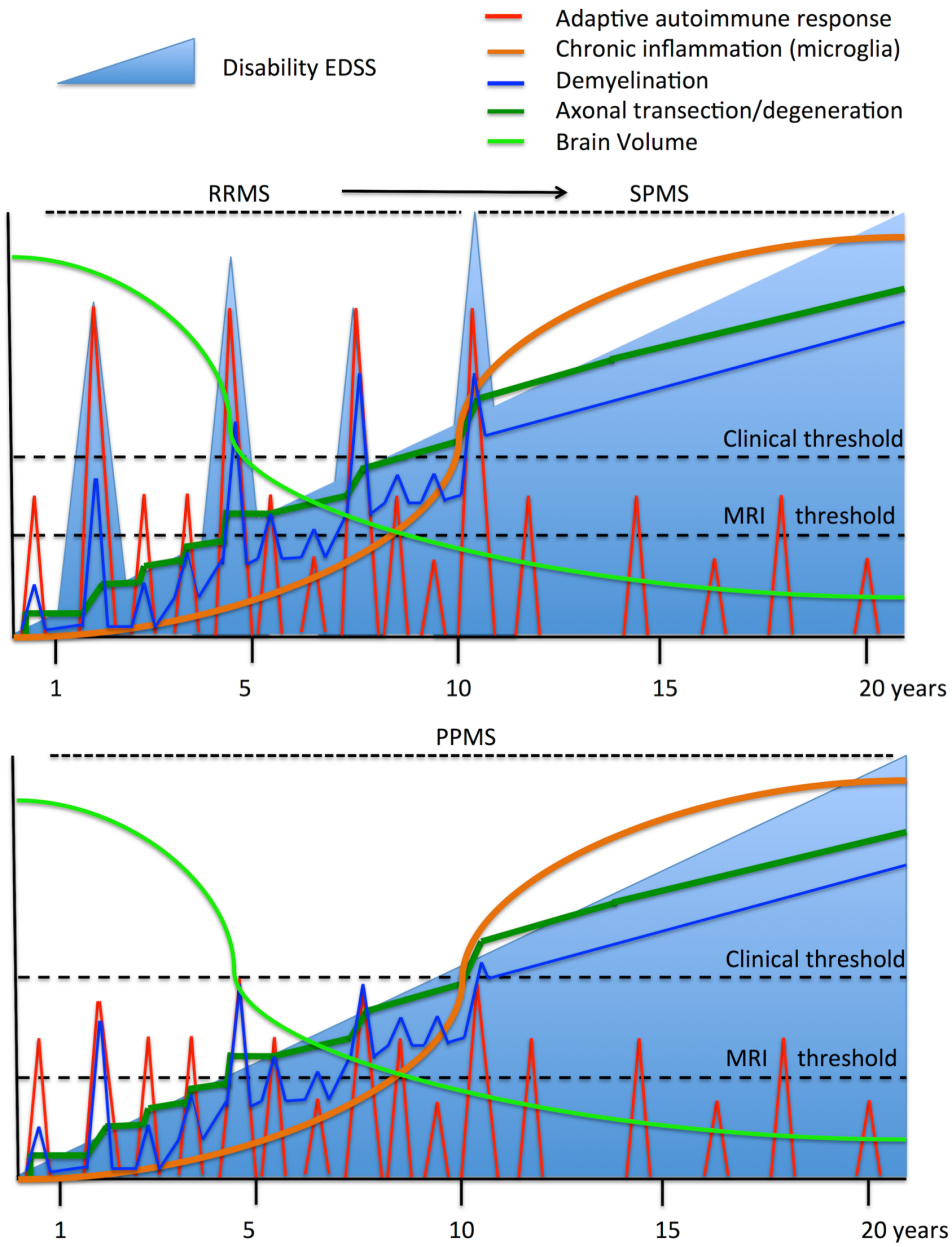
Dynamics of the clinical and pathogenic processes in MS. The upper panel shows the evolution of relapsing-remitting MS (RRMS) and its transition to secondary-progressive MS (SPMS), while the bottom panel shows the evolution of primary-progressive MS (PPMS). The autoimmune process starts in the peripheral immune system, inducing episodes of CNS inflammation (red line) that subsequently provokes demyelination (blue line) and then axon degeneration (dark green line). Although inflammation and demyelination may experience remissions, axon degeneration accumulates over time, as does chronic compartmentalized inflammation (orange line). If inflammatory infiltrates affect eloquent CNS regions and exceed damage thresholds, they manifest as clinical relapses. Alternatively, when cumulative axon degeneration surpasses the capacity of the functional CNS reserve, permanent neurological disability arises (light blue line) and there is a transition to the progressive disease. The decrease in brain volume over time is more severe at the beginning of the disease, in parallel with more intense inflammatory activity, and it continues steadily as the disease evolves. PPMS follows the same processes but the inflammatory relapses are not translated into clinical relapses, either because they are less frequent, less severe or they affect silent (non-eloquent) areas. Only when axon degeneration reaches a clinical threshold is disability manifested as progressive. Therefore, there are no differences between SPMS and PPMS except for the relative clinical impact (relapses) of acute inflammatory activity.

CNS damage in MS is caused by acute inflammatory infiltrates (composed of lymphocytes and macrophages) and chronic compartmentalized inflammation (driven by activated microglia/macrophages in the CNS parenchyma and meningeal inflammation), as well as by axon degeneration (e.g., axon degeneration due to the demyelination, neuronal pruning or cell death, or by transynaptic degeneration: Fig 1) [7, 12, 13]. Recent pathological studies have shown that acute inflammatory damage predominates in the early stages of MS, while chronic inflammation (smoldering plaques) prevails at later stages, suggesting an evolution from peripheral autoimmune damage to chronic CNS inflammation [14].

Long-term cohort studies have shown that the progressive course of MS develops after a given threshold of disability is reached, defined by a score above 4.0 in the Expanded Disability Status Scale (EDSS). Hence, inflammatory and neurodegenerative processes appear to be to some extent independent [15], supporting the two-stage disease hypothesis. Alternatively, genetic susceptibility always appears to be related with immune system dysfunction and not with disease course [16]. Moreover, pathological inflammation is always detected in MS, in all phases of the disease, with a predominantly adaptive immune response in the early stages of the disease and a mainly innate immune response (compartmentalized inflammation) at later stages [13, 14]. Therefore, while the evolution of MS seems to be the result of the interplay between acute inflammatory relapses, chronic inflammation in the CNS, and the degeneration of axons and myelinated cells, each process could have a distinct influence on the different patient subgroups and at different stages of the disease, consistent with the single-disease hypothesis. On top of this, we should also consider the role of functional CNS adaptation or the functional reserve at disease onset. There is little structural damage at disease onset and thus, the breakdown of functional CNS compensation and disability increases from a certain damage threshold onward [12].

In RRMS, the main drivers of clinical disability include demyelination, the blockage of axonal conduction and acute axon transection, whereas axon/neural loss is the main factor underpinning permanent disability during the progressive phase [17]. While demyelination is frequently reversible during the RRMS phase, complete recovery is rarely achieved [18]. As such, demyelination in RRMS can be expected to lead to a certain degree of permanent damage that accumulates after each relapse and that exacerbates the effects of acute axon transection.

In order to improve disease management, models of CNS damage and disease dynamics could be useful to stratify patients with similar degrees of disability, as well as to evaluate the benefits of therapies in different patient sub-types [19]. Complex diseases can be modeled on different biological scales, from the molecular level (e.g., genetic networks and signaling pathways) [20–22], to the cellular level [4] where the cell is considered as the basic unit that integrates all the molecular information (e.g., lymphocyte dynamics), or to the tissue or whole body level [5]. We previously developed a mathematical model of the autoimmune response at the cellular level that reproduces the dynamics of MS relapses [4, 23]. As a result, cell interactions were seen to particularly affect tissue dynamics at the functional level, influencing the immune response or CNS neurodegeneration. Hence, modeling at the cellular level may bring us closer to the clinical phenotype.

Here, we have tested the hypothesis of dynamic CNS damage which states that the course and heterogeneity of MS can be generated through a specific pathogenic mechanism, namely autoimmune attack on the CNS in association with chronic inflammation, the severity and timing of each process producing the diversity in the patient subgroups. We assessed to what extent the progression of CNS damage in MS is a dynamic process that commences at the onset of the disease, even in RRMS. For simplicity, our model only included inflammation, demyelination and axon loss at a general level, without entering into specific mechanistic details, such as the role of the adaptive and innate immune responses, or feedback from CNS damage to the autoimmune process. Consequently, our model does not formally exclude other alternative hypotheses (e.g., the two-stage disease or the inside-out hypothesis).

## Results

### Modeling the dynamics of CNS damage in MS

**Fig 2:**
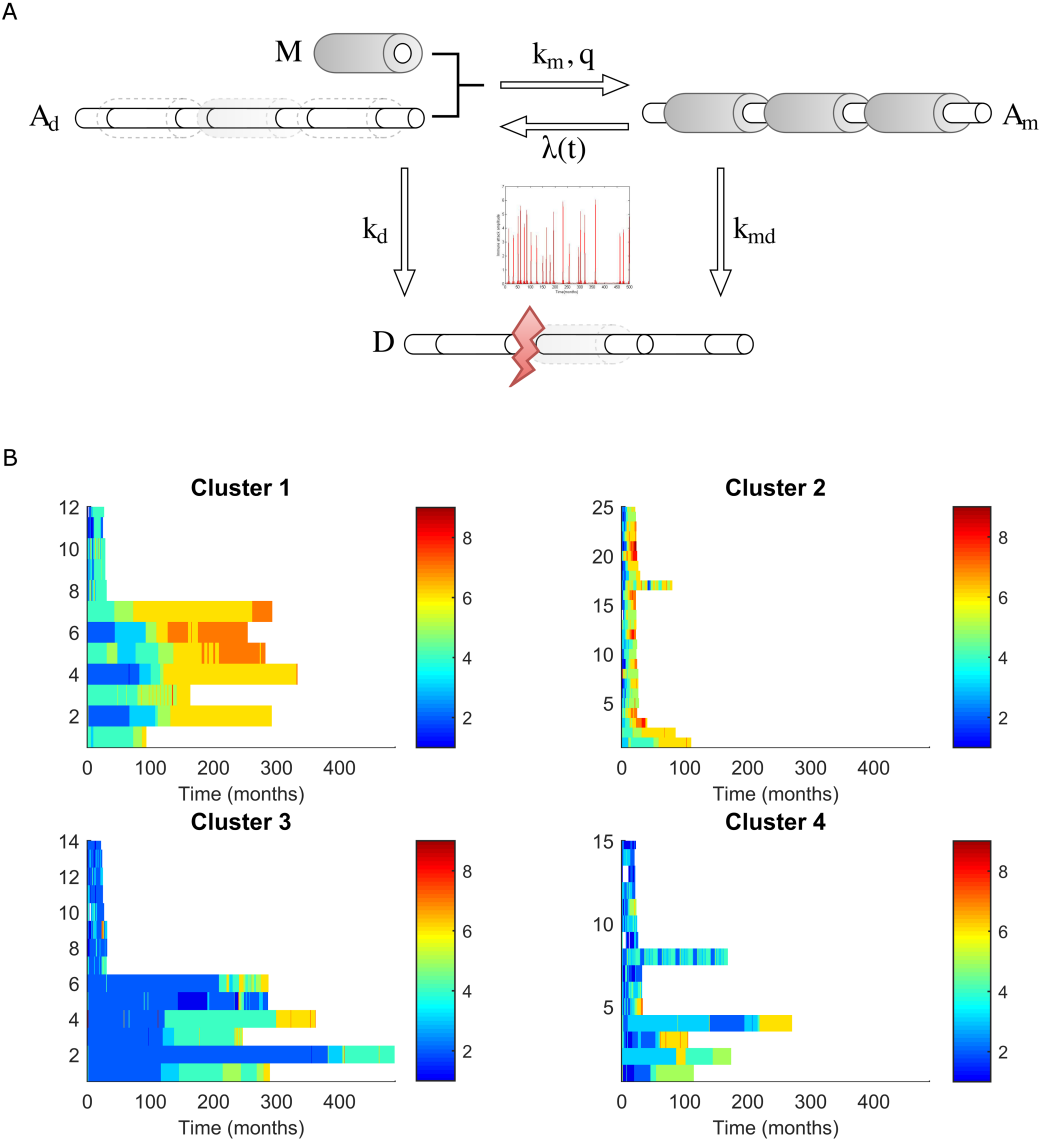
The ODE model of CNS damage in MS. A) The model represents the volume occupied by axons and myelin: Right, the healthy CNS is composed of myelinated axons (*A*_*m*_); Center, inflammatory attack is represented by the time-dependent parameter λ(t), which arises from the Generalized Extreme Value (GEV) distribution of the EDSS time-series, producing either demyelination (right) or degeneration of axons (bottom); Left, demyelinated axons (*A*_*d*_) can be remyelinated with myelin produced by oligodendrocytes (*M)*, as a function of the parameters *km* and *q*; Bottom, myelinated or demyelinated axons can be lost by either acute axon transection or degeneration (*D)*, according to the parameters *kmd* or *kd* respectively. B) Clustering MS patients based on the EDSS time series. The horizontal axes correspond to the time in months (maximum = 16 months), while the vertical axes correspond to the patients. Each line represents the EDSS of a given patient over time, using a color scale to reflect the EDSS. Clusters 1 and 2 include patients that maintain an intermediate short term EDSS and that reach a high EDSS in the long term. Cluster 3 includes patients that maintain a low short term EDSS and that achieve an intermediate EDSS in the long term. Cluster 4 represents a more heterogeneous group.

We developed a mathematical model based on ordinary differential equations (ODEs) to study the dynamics of CNS damage in MS, a model that contemplated the dynamics of myelinated (healthy) and demyelinated axons, axon loss (acute transection or delayed degeneration) and demyelination/remyelination as a consequence of autoimmune attack (see Materials and methods: Fig 2A). We defined the parameters of the model through a literature search (S1 Table) and then, by fitting it to the changes in EDSS in a retrospective, longitudinal cohort of MS patients with a long (up to 20 years) follow-up. The results were validated in a second prospective cohort with a shorter follow-up (3 years), and brain volume (BV) quantified by MRI was used in the validation cohort to improve the fit of the cell damage in the model to the clinical phenotype (EDSS time series). In addition, to decrease the complexity of the clinical phenotype, the data were grouped using a non-supervised clustering approach (see below).

### Clustering of the EDSS time series

The EDSS time-series from the retrospective longitudinal cohort was clustered using a modified k-means clustering method and subsequently, we analyzed each cluster as a single dataset to estimate the distinct parameters. We identified four clusters of patients that corresponded to the different levels of disease severity (EDSS) and disease subtypes (relapsing vs progressive disease: Fig 2B). Thus, 86% of progressive MS cases (SPMS or PPMS) were included in Cluster 1 or 2, whereas 66% of relapsing MS (RRMS) were grouped into Cluster 3 or 4 patients (Table 1). We validated this clustering using a cohort that consisted of an EDSS time-series with a three-year follow-up (see Materials and methods), producing a similar grouping into four clusters.

**Table 1:** Proportion of each MS subtype in the clusters identified in the discovery cohort.

# of cases (%)*	RRMS	SPMS	PPMS	All
**Cluster 1**	1 (7%)	7 (17.5%)	3 (27%)	11
**Cluster 2**	1 (7%)	16 (40%)	8 (73%)	25
**Cluster 3**	6 (43%)	8 (20%)	0 (0%)	14
**Cluster 4**	6 (43%)	9 (22.5%)	0 (0%)	15
**All clusters**	14 (100%)	40 (100%)	11 (100%)	65

*One patient was not ascribed to any of the clusters and was excluded from the analysis, giving a total of 65 patients.

### Simulations with the model reproduce the observed MS phenotypes

**Fig 3:**
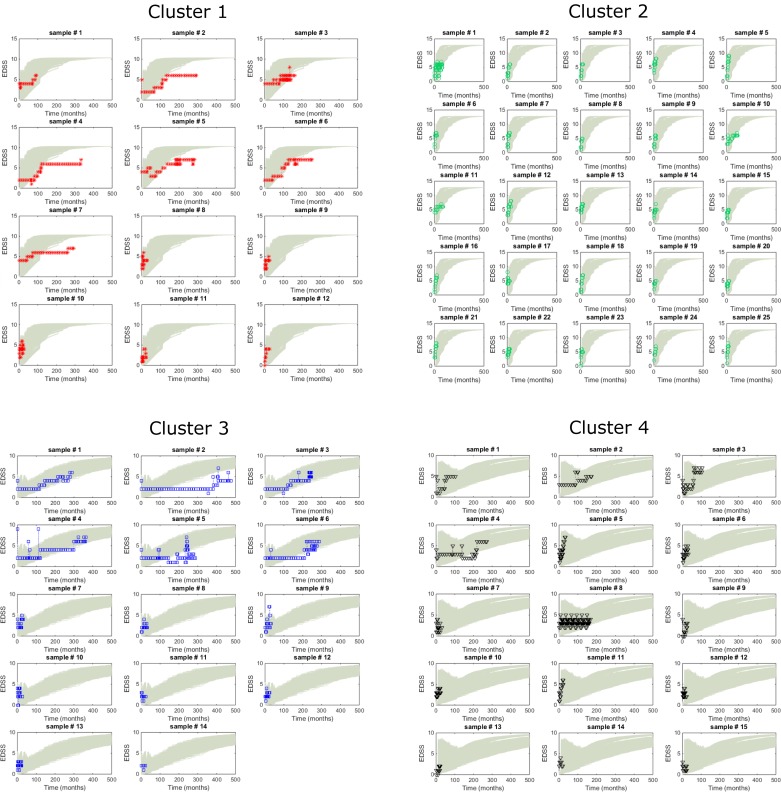
Comparison of the EDSS time-series and model simulations. Comparison of the EDSS scores from all the patients in the longitudinal cohort as a function of their cluster (each patient from the discovery cohort is shown separately and identified with a cluster-specific color: red, cluster 1; green, cluster 2; blue, cluster 3 and black, cluster 4) and in accordance with the model’s predictions (grey lines, each line corresponds to an individual simulation).

Simulations with the model were compared with the experimental EDSS time-series using the 10 parameter sets with the best data fit (lowest objective function values, see Materials and methods: S1 Fig) and 100 random (*t*) inputs of the cluster-specific EDSS characteristics. The EDSS time-series closely overlapped the simulations for each cluster, confirming that the model could reproduce the diversity of MS phenotypes (Fig 3).

To evaluate the quality of the predictions for each cluster at the clinical level, we calculated the probability of reaching key clinical milestones, such as EDSS 4.0 or 6.0 [15]. The event of interest was calculated as the ratio of patients with an EDSS higher than the value of interest at each time point. Again, the EDSS time-series were contained within the model’s simulations, confirming the ability of the dynamic model of CNS damage to reproduce the different trajectories towards a milestone of disability (S2 Fig).

**Fig 4:**
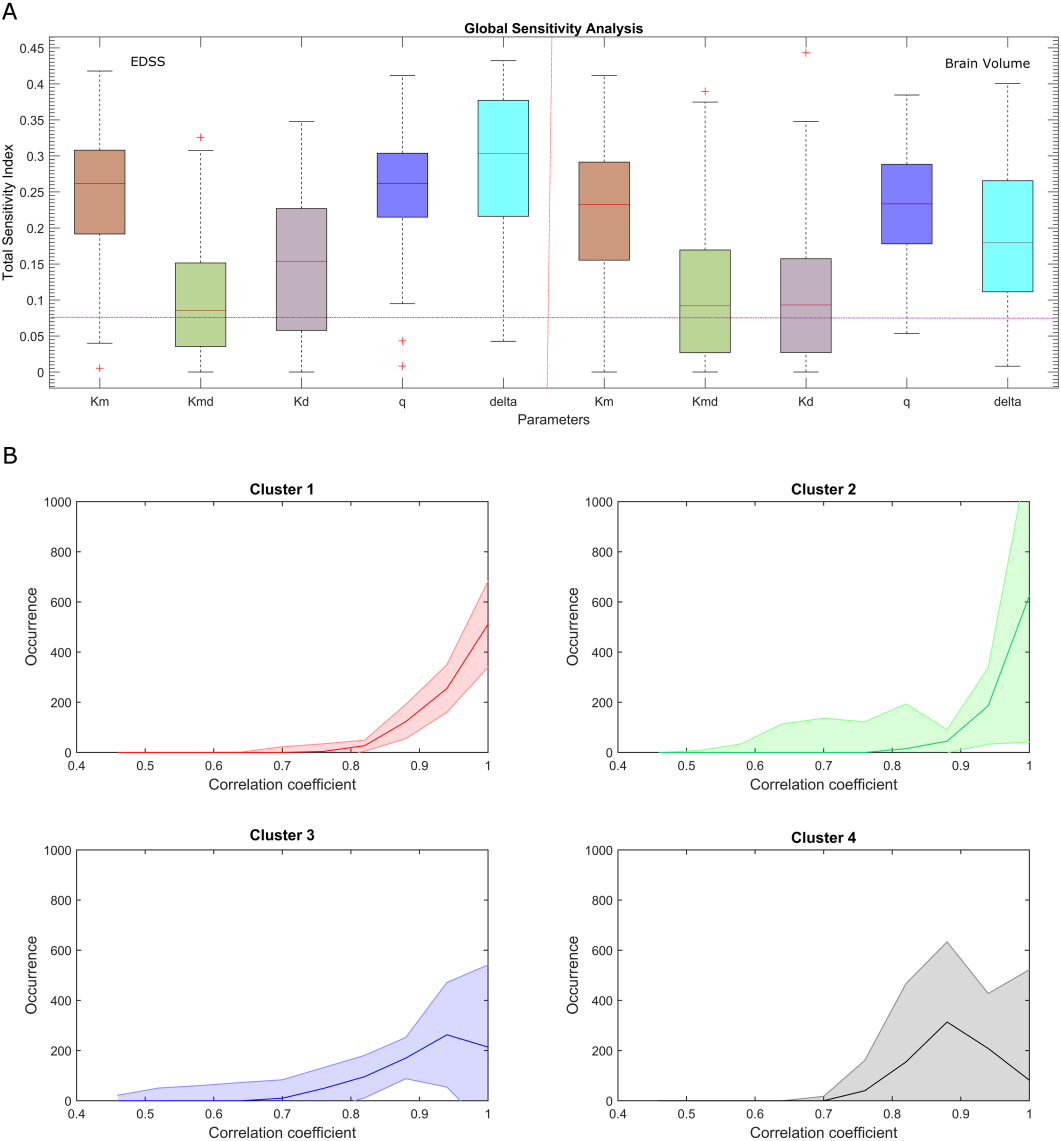
Sensitivity analysis of the model’s parameters. A) A sensitivity analysis showing the effects of the uncertainties in parameters on the model’s behavior (output variables: EDSS and brain volume—BV). The extended version of a Fourier amplitude sensitivity test (eFAST) was used to quantify the relative importance of the input factors. Pink line indicates the sensitivity level of a dummy parameter that does not occur in any of the equations. Sensitivities below this line should not be considered significantly different from zero. B) Simulations of brain volume in MS. Median occurrence and interquartile range (IQR) for the correlation coefficients between the experimental and simulated BVs in the validation cohort. Pearson correlation coefficients: cluster 1, 0.93; cluster 2, 0.94; cluster 3, 0.86; and cluster 4, 0.87.

In order to investigate the influence of the parameters on disease dynamics, we compared their sensitivity profiles (Fig 4A). We found that the remyelination-related coefficients *k*_*m*_ and *q* have an important influence on the demyelination coefficient *k*_*md*_, which is consistent with the fact that disrupted myelin and impaired remyelination are processes responsible for MS progression [11]. Global sensitivity indices were calculated for the model read-outs (EDSS and BV, the latter measured by MRI), show notable differences in the demyelination coefficient *k*_*d*_ (p = 0.0027), as for δ (p = 1.81E-09) and to a lesser extent *q* (p = 0.0252). The sensitivity of the parameters with respect to EDSS or BV were almost equivalent, suggesting that there is no difference in the use of either. Thus, there is no parameter that is non-identifiable due to the type of data, although the goodness of fit will indicate that which is best used for modeling.

### The dynamics of brain atrophy

We tested if the model could also reproduce the dynamic changes in BV of the validation cohort. As expected, there was a negative correlation between the EDSS and BV time-series in the prospective cohort (S3 Fig). As indicated in the Materials and Methods, we estimated the coefficients necessary to connect the values observed to the simulated read-outs specific to the allocated cluster (note that we did not recalculate the parameters but rather, we used the parameter estimated from the discovery cohort), and we then adjusted the age-dependent initial value *A*_*mi*_(t = 0). For each of the clusters we compared 1000 simulated time series (100 simulations for the 10 selected parameter sets) of the BV (*V*_*s*_(t)) using the normalized BV values from the MS patients. The correlation coefficient was calculated for each combination of experimental and simulated time series. For each patient, we generated a distribution of the correlation coefficients between the experiments and simulations (Fig 4B), and we observed a significant correlation between the experimental and simulated BV (median correlation coefficients: cluster 1, 0.93; cluster 2, 0.94; cluster 3, 0.86; and cluster 4, 0.87).

### MS subtypes are the consequence of different dynamics of the same pathogenic process

**Fig 5:**
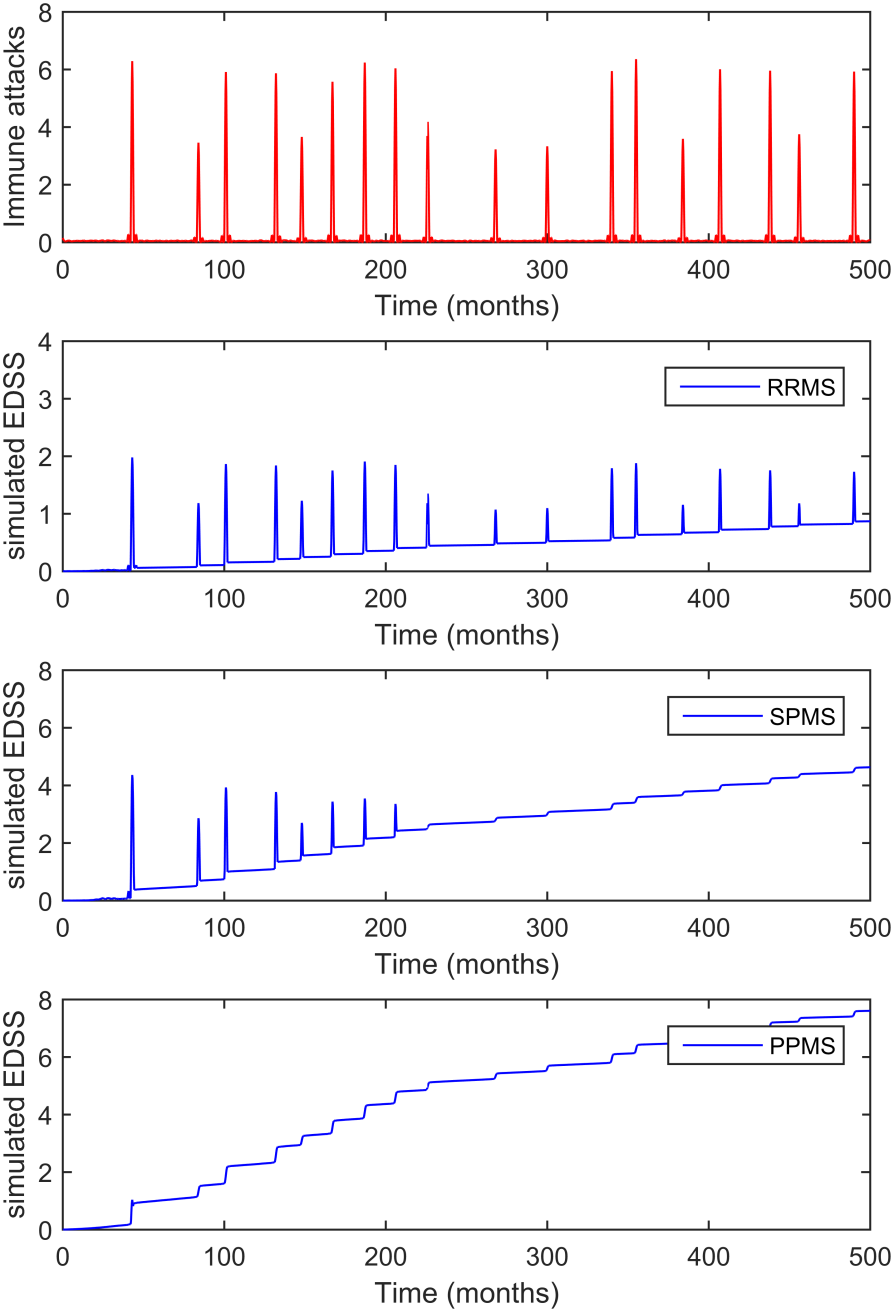
Simulations of the model that reproduce the MS subtypes. The top panel shows an example of the dynamics of autoimmune attacks (derived from the T cell model and adjusted for the EDSS distribution), and the bottom panels reproduce the dynamics of the EDSS in function of the fine-tuning of the parameters defining axon degeneration and de/remyelination, reproducing the RRMS, SPMS and PPMS disease courses, respectively. In the model, each increase in the EDSS is the consequence of an inflammatory attack, yet in the progressive phenotypes (SPMS and PPMS), they are observed as small and discrete increases in the EDSS.

We first evaluated whether the model could simulate the diversity of disease subtypes (RRMS, SPMS and PPMS) and their severity. Simulations were performed with the same inflammatory input (*t*) but with different sets of parameters, and these parameters were then explored manually. Simulations reproduced the different disease subtypes (Fig 5), whereby the different disease courses -RRMS, SPMS and PPMS- were reproduced by changing the parameters of the model given that the inflammatory part of the disease remained the same. The overlap between the inflammatory inputs and the EDSS course indicated that each significant incremental change in the EDSS is the result of a specific immune attack (relapse), whereas the progressive increases in the EDSS result from changes in the parameters of chronic inflammation and neurodegeneration (progressive phase).

### Progressive and relapsing MS are inversely dependent on the rate of axon degeneration and the capacity for remyelination

**Fig 6:**
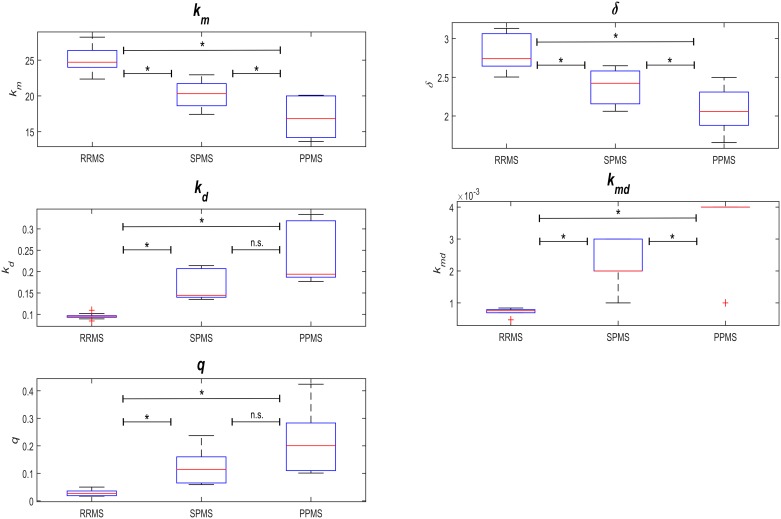
Model parameters for the studied disease subtypes. Parameter distribution for the different disease subtypes were analyzed through pairwise Wilcoxon tests (S2 Table).

By running simulations of our model and comparing the results with the EDSS time-series of the longitudinal cohort, we tested whether the model reproduced the phenotypes of the four patient clusters when the parameters were modified, albeit maintaining them within the biological range (Fig 1 and S1 Table). To identify the parameters that would reproduce the different MS subtypes (RRMS, SPMS and PPMS), we calculated the number of patients of each subtype in each cluster and expressed this relative to the total number of patients of that subtype in the discovery cohort. These ratios allowed us to compute linear combinations of the cluster-specific parameter sets (the 10 combinations of parameters with the 10 lowest objective values in terms of function) in order to define MS subtype-specific parameters sets (S1 Fig). We then compared these values between the three MS phenotypes (using a pairwise Wilcoxon test), and we found the *Km*, *Kmd*, *Kd* and *δ* values to be significantly different between all three MS subtypes, whereas *q* only differed significantly between the RRMS and PPMS subtypes (Fig 6; S2 and S3 Tables).

By definition, the biological meaning of the model parameters *K*_*m*_ and *δ* are related to the rate and capacity of remyelination, respectively, while *K*_*d*_ and *K*_*md*_ define the rates of irreversible axon degeneration for demyelinated and myelinated axons, respectively. The subtype-specific parameters estimated above indicated that the characteristic accumulated and irreversible disability of the progressive MS subtypes (PPMS and SPMS) is associated with higher rates of axon degeneration. This association was revealed by the significant monotonic increase in *K*_*d*_ and *K*_*md*_ from RRMS to SPMS and PPMS, and the lower rate and capacity of remyelination (a significant monotonic decrease in *K*_*m*_ and *δ* from RRMS to SPMS and PPMS: Fig 6 and S2 Table). However, the degeneration of chronic demyelinated axons is not a key feature distinguishing the RRMS phenotype, consistent with recent pathological studies [17, 24, 25].

A statistical analysis of the model’s parameters between the disease subtypes suggested that the greater resilience to CNS damage and disability in the relapsing subtype (RRMS) relative to progressive MS (SPMS and PPMS) is related to the lower rate of axon degeneration, and more rapid remyelination. The higher remyelination capacity in RRMS is reflected by a higher *K*_*m*_ (p = 0.0002) and *δ* (p≤0.001), and the lower rates of axon degeneration in RRMS are indicated by lower values for *K*_*d*_ (p = 0.0002) and *K*_*md*_ (p≤0.0001: Fig 6 and S2 Table).

### MS is a progressive disease from the onset, with superimposed relapses

Another relevant question is whether the transition from relapsing to progressive MS is a dynamic process that simply involves the same biological processes or whether any additional biological events must be invoked to explain its dynamics, e.g., the effects of neurodegeneration on axons [26]. As such, we analyzed the transition from the relapsing to the progressive phase in terms of the changes to the variables within the model. The model readout (EDSS) is defined as the maximum of *Ad* and *D*, where *D* refers to irreversible axon degeneration. From the equations, one can see that *D* is always an increasing monotonous function (axon transection), while *Ad* (demyelinated axons) is the variable derived from inflammatory relapses when the condition *Ad* > *D* is met (otherwise, if *D* > *Ad*, the effect of *Ad* is masked by *D*). Hence, disability in RRMS can be characterized by the impact of demyelination due to relapses whereas in the progressive phase of MS the impact of axon degeneration (D, transected axons) is stronger than the impact of demyelination (*D* > *Ad*). Thus, the nature of the model defines the transition from the relapsing to the progressive phase of MS as a dynamic evolution from *Ad* > *D* to *D* > *Ad*.

We found that the PPMS phenotype was also reproduced by the model (Fig 5), a phenotype that occurs when *D* > *Ad* at all time points. This criterion could be met when the rate of axon degeneration is high (large values of *K*_*d*_ and *K*_*md*_) and when remyelination fails (*K*_*m*_ and δ are small, see Fig 2), particularly given that myelinated axons are less prone to degeneration than demyelinated axons (*K*_*md*_ << *K*_*d*_). Thus, our model of CNS damage in MS supports the concept of MS as a single and progressive disease, with individual heterogeneity based on differences in dynamics, a notion consistent with pathological findings [27].

A central question is whether neurodegeneration in MS is a process that is independent of inflammation, appearing at later phases in a damaged CNS, or if a single progressive process is at play that commences at disease onset and that combines both biological processes to a different extent over time. Simulations of the model reproduced all the MS phenotypes when both inflammation and degeneration commenced at disease onset (Fig 5). In addition, the model assumed a direct effect of adaptive immune system attacks (the independent parameter *λ(t)*) on demyelination-related relapses and neurodegeneration, as well as an effect of chronic immune activation (through parameters *k*_*d*_ and *k*_*md*_) on neurodegeneration. Accordingly, our simulations support the concept that neurodegeneration starts from the beginning of the disease and progresses at different speeds in different patients. However, our model did not explore other alternative hypothesis.

## Discussion

In this study, we tested the dynamic CNS damage hypothesis of MS, and whether all the disease phenotypes can be reproduced by the participation of the same mechanisms operating at different intensities and over different time scales: autoimmune inflammation followed by axon loss and de/remyelination. Our simulations support the hypothesis that MS is a single disease with very heterogeneous phenotypes and they suggest a different contribution of each process to the phenotype. The presence of irreversible axon degeneration at early disease stages would appear to be mainly due to the higher rates of degeneration (transection) of myelinated axons and to a lesser extent, to a weaker capacity for remyelination. A build-up of axon degeneration is the basis of the progressive phenotype, even during early disease stages like those of RRMS. Conversely, increased resilience in both the rates of axon degeneration and in the efficiency of remyelination at early stages of the disease are the basis of the RRMS subtype. These results provide a theoretical framework to study the contribution of such pathogenic processes at the experimental level, as well as for the design of therapeutic strategies for MS. However, our model does not rule out alternative hypotheses, such as the inside-out hypothesis, the two-stage hypothesis or the influence of a deteriorated autoimmune response (e.g., epitope spreading, antigen presentation in the damaged CNS). Hence, we can only state that the dynamic CNS damage hypothesis of MS is consistent with the phenotype observed, while we cannot formally rule out other explanations.

In order to use clinical data to fit the parameters to our model, we performed a clustering analysis to limit the heterogeneity of the data. Non-supervised clustering yields four clusters that best group the data and that reproduce the main characteristics used clinically to stratify patients: namely the disease subtype (relapsing or progressive) and disease severity (commonly defined as the time to reach a milestone like EDSS 4.0 or 6.0). It is striking that a simple approach such as a clustering analysis does not segregate the MS phenotypes into the three classic subgroups, supporting the current concept that RRMS-SPMS-PPMS represent a continuum with different levels of disease activity and superimposed relapses, as proposed recently [12]. Based on this approach, our model has been optimized to match each of the clusters and such a grouping may aid patient stratification at the time of tailoring therapies based on disease course. As such, new prospective clinical studies classifying patients into one of the four clusters and modeling the trajectory of each group based on this ODE model should provide evidence of its clinical utility for patient stratification.

Our model may have implications for the development of new therapies for MS. Pathological studies have shown that all pathogenic processes are in place from the onset of the disease [14] and they demonstrated the key role of acute axon transection due to autoimmune relapses [28]. Based on these concepts, it has become highly desirable to obtain “no evidence of disease activity” (NEDA) from the early stages of the disease [29]. Our model supports this assumption, although such predictions must be demonstrated in clinical trials or by ruling out other alternative hypotheses. This is important because the model shows that CNS damage accumulates from disease onset and that once it reaches a given threshold, a small increase in damage has a significant impact on disability. This can be explained by depletion of the functional CNS reserve, hindering remyelination and impairing axon conduction due to demyelination [12]. Although there is currently much interest in remyelination therapies, our model suggests that these may only be of value within specific time windows of disease evolution (e.g., RRMS). However, remyelination was not fully analyzed with our model and thus, our results should be considered preliminary. Finally, our model shows a key role of axon degeneration in defining the MS phenotype, consistent with pathological evidence. As such, developing neuroprotective or regenerative therapies should hinder the advance of disability [30].

Our approach has some caveats. At the formal level, this study offers support for the dynamic CNS damage hypothesis of MS but it does not formally exclude alternative hypotheses, such as the two-stage hypothesis or the inside-out hypothesis. Considering the lack of quantitative biological data regarding these biological processes, we approached the parameter search by fitting the ODE model to the clinical phenotype (experimental EDSS time-series) and we then checked whether such parameters were in the range of biological processes. Therefore, our results should be considered more a qualitative than quantitative model of CNS damage in MS. In addition, we have not modeled all the pathogenic processes that can damage the brain in detail, such as the feedback of CNS damage to autoimmune processes, chronic microglial activation, meningeal inflammation, cortical plaques, or specific neurodegenerative processes. Future studies and more quantitative data will allow such a level of detail to be added, and enable more specific and quantitative models to be developed. On a more positive note, we were able to model the phenomena using relatively few parameters, which means that the constitutive equations are capable of capturing MS demyelination and neuroaxonal events.

In summary, our study indicates that the pathogenic processes that drive autoimmune damage in the CNS can produce all the distinct MS subtypes and explain the clinical heterogeneity in patients. However, while each phenotype requires specific parameters to be fulfilled, it appears that there is a distinct contribution of each biological process to the different disease stages (perhaps reflecting different genetic susceptibility and environmental exposure). Therefore, our model supports the notion that MS and its phenotypes can be explained as an autoimmune process, arguing in favor of the dynamic CNS damage hypothesis of MS. This hypothesis has implications for the development of new therapies and patient monitoring. Principally, it means that MS should be considered and treated from the onset as a progressive disease, with a focus on preventing CNS damage, and on avoiding reaching the thresholds associated with the progressive course of the disease and more severe disability.

## Materials and methods

### Ethics statement

All the patients were recruited by their neurologists after obtaining their signed informed consent. The IRB of the Hospital Clinic, Charite University, Karolinska Institutet, University of Zurich approved the study.

### Patients

The discovery cohort was a retrospective longitudinal cohort that included 66 MS patients from the Hospital Clinic of Barcelona, Spain (iTEM database) and from the Hopital Civil de Lyon, France (EDMUS database, data provided by Prof. Christian Confavreux: the raw data for the EDSS time-series are provided in the S1 File). The time-series of the cohort included EDSS values during a follow-up of 5 to 20 years. The disease subtypes at the end of the follow-up were: 22% RRMS, 60% SPMS, and 16% PPMS. The validation cohort was a prospective cohort of 120 MS patients from the Hospital Clinic of Barcelona, with annual clinical (EDSS) and MRI assessment over three years (raw data for the EDSS and BV time-series are provided in the S2 File). More details of this cohort can be found elsewhere [31].

### MRI volumetric dataset

MRI’s were acquired on a 3T Magnetom Trio scanner (Siemens, Erlangen, Germany), using a 32 channel phased-array head coil. A 3-dimensional structural T1-weighted Magnetization-Prepared Rapid Gradient Echo (T1-MPRAGE) was used to compute all the volumes in this study and a 3-dimensional Fluid-Attenuated Inversion Recovery (FLAIR) was used to manually achieve lesion segmentation. The T1-weighted MPRAGE sequence was acquired with the following parameters: TR = 1970 ms, TE = 2.41 ms, TI = 1050 ms, flip angle = 9, 208 contiguous sagittal slices with voxel size = 0.9 x 0.9 x 0.9 mm3, matrix size = 256 x 256. The FLAIR sequence was acquired with the following parameters: TR = 5000 ms, TE = 393 ms, TI = 1800 ms, 208 contiguous sagittal slices with voxel size = 0.9 x 0.9 x 0.9 mm3, matrix size = 256 x 256. The FLAIR image registered to T1 was used to manually segment the lesions. Subsequently, the lesion mask obtained was used to create a healthy-like T1 and improve the following steps. Finally, the T1 was segmented and the normalized BV was calculated using SIENA.

### Clustering of EDSS data

We used a modified k-means algorithm to cluster the patient’s EDSS time-series from the discovery cohort based on their complete EDSS time-series. First, we normalized the EDSS data at each time point to the maximum and imputed the missing values using the K-Nearest Neighbor method. Since k mean clustering is sensitive to the choice of the initial partition, we ran it multiple times with random starting points, and using different k values between 3 and 8. The center of the clusters were obtained using fuzzy c-mean clustering and through this method, we identified clusters that group all patients in the dataset. The number of clusters was quantified using the average silhouette approach, which provided the best result at k = 4. We obtained the clusters that better grouped the dataset of the discovery cohort, without pre-specifying any number of clusters (e.g., 3 clusters to group RRMS, SPMS and PPMS).

To test if the clustering obtained from the long-term follow-up datasets (i.e.: the discovery cohort) could cluster clinical datasets when only short-term follow-ups are available (e.g., two-three years of annual EDSS data), we evaluated if the four clusters identified in the discovery cohort were also extracted from the validation cohort. As such, we repeated the clustering process on the validation cohort with the restriction that the number of clusters should be the same as that defined in the discovery cohort (n = 4). We calculated the ratio of misclassified patients for each of these 4 clusters in the validation cohort compared to the discovery cohort as the error rate. We found that the error rate of the clustering using only a 2-year follow-up was 16.51% ± 6.02 (the maximum error rate belonged to cluster 3 and the minimum rate to cluster 2). Hence, based on these results we established that classification or assignment to EDSS clusters can be achieved based on short-term EDSS observations.

### Distribution of ΔEDSS to define the inflammatory input

Incremental changes in disability (ΔEDSS) were calculated using the current confirmed definition of the progression of disability based on an increase ≥1 point in the EDSS three months apart [2]. Such ΔEDSS may be due to clinical relapses or disease progression. For the EDSS time-series, the time intervals (ΔT) between consecutive ΔEDSS events (pulses) were counted and using MATLAB (allfitdist custom script), we tested different statistical models to identify that which best described the distribution of the ΔT values. The frequency of the ΔEDSS events in 76% of patients can be approximated by the inverse Gaussian distribution and the remaining 24% by the distribution of Generalized Extreme Values (GEV: comparable statistical models representing rare events). Furthermore, we took the median values of ΔT for each of the patients and analyzed the resulting distribution of all of them. The best approximation of the resulting distribution was the GEV model (cumulative distributions versus data: S4 Fig). The simulated GEV distribution of the ΔT frequencies belongs to a category of extreme events of underlying processes and it was further used in the ODE model with the parameters of distribution, defined separately for each patient cluster.

### Mathematical model: Biological and clinical assumptions

MS is an autoimmune disease in which the activation of auto-reactive T cells induces chronic activation of the innate immune response and focal CNS damage, the latter manifested as demyelination and axonal loss (Fig 1) [1, 7, 32]. Mechanisms that drive peripheral immune tolerance and brain immune privilege can shut down the immune attack in the short term, although relapses occur that exacerbate demyelination and axon loss. We assume that remyelination fails after some time, contributing to the steady loss of axons and the chronic compartmentalized inflammation that ultimately leads to neurodegeneration [28]. However, inflammation persists throughout the disease and it evolves from being orchestrated in the peripheral immune system to being compartmentalized in the CNS [33, 34]. During the early phases of the disease, immune-mediated demyelination and acute axon transection dominate, while the progressive phases are characterized by compartmentalized CNS inflammation and degeneration of demyelinated axons due to oxidative stress, energetic failure, loss of trophic support in the oligodendrocyte-axon unit and the development of a glial scar [6, 25, 35]. We took these three processes (inflammatory attack, demyelination/remyelination and axon loss) into account at the cellular level to model MS and reproduce the clinical phenotypes observed. We kept our model as simple as possible using the most basic processes described in MS, and avoided modeling other processes for which there is still insufficient quantitative data for them to be modeled (e.g., specific inflammatory processes or oligodendrocyte loss).

The healthy CNS is composed of neurons and their myelinated axons, and for simplicity we do not consider the volume of glial cells (e.g., astrocytes and microglia) or the somas of oligodendrocytes. Myelin is damaged during the course of autoimmune attack (via pro-inflammatory cytokines, antibodies), oxidative stress or energy failure [36, 37]. Yet because extensive remyelination may occur in the early to mid-phase of MS [18], we assume that demyelination/remyelination events will follow the dynamics of the autoimmune attack until remyelination mechanisms are unable to compensate for the loss of myelin, which occurs in conjunction with the clinical transition to the progressive phase [24]. By contrast, axon damage (acute transection or degeneration) does not follow autoimmune dynamics but rather, it accumulates due to the poor regenerative capacity of the CNS [12].

Activation of the adaptive immune system in MS drives the migration of lymphocytes and monocytes into the CNS, damaging the CNS parenchyma. Inflammatory infiltrates are present in all forms of MS, including PPMS, albeit in different proportions and with distinct temporal profiles for each disease subtype [14]. Both reversible CNS damage (demyelination with axon preservation) and irreversible CNS damage (permanent demyelination and axon transection/degeneration or neuronal loss) lead to a wide range of neurological disabilities (as measured with the EDSS [38]).

We developed an ordinary differential equation (ODE) model that aimed to reproduce the healthy brain (myelinated axons, *A*_*m*_), the MS brain (the combination of *A*_*m*_, demyelinated axons, *A*_*d*_, remyelination capacity, *M*, and axon degeneration, *D*). We represent the fraction of the BV occupied by myelinated and demyelinated axons as *A*_*m*_ and *A*_*d*_, respectively, and we define the capacity for remyelination as *M* (the ability of oligodendrocytes to produce all the myelin required in a healthy adult brain). Our model describes three main processes in MS that are involved in CNS damage: immune attack (via either inflammatory infiltrates or chronic compartmentalized inflammation); demyelination/remyelination; and axon degeneration.

$$\frac{dA_{m}}{dt}=\;k_{m}A_{d}M\;-\lambda \left(t\right)A_{m}-\;k_{md}A_{m}$$ (1)

$$\;\frac{dA_{d}}{dt}=\;-k_{m}A_{d}M+\lambda \left(t\right)A_{m}-\;k_{d}A_{d}$$ (2)

$$\;\frac{dM}{dt}=-k_{m}A_{d}M+\delta \;A_{d}M^{q}$$ (3)

$$\frac{dD}{dt}=\;\;k_{d}A_{d}+\;k_{md}A_{m}$$ (4)

The first term on the right-hand side of the three equations represents the remyelination process, at a rate of *k*_*m*_. In turn, axons become demyelinated at a basal rate *k*_*md*_. The capacity of myelination (*M*) is depleted by the demyelination process and restored by the production of myelin segments by oligodendrocytes. *M* is defined as the amount (volume) of myelin that can be produced in a healthy adult brain and it is used to model the remyelination capacity modulated by *k*_*m*_. Up to 40 myelin segments are produced by a single oligodendrocyte and those segments can be extended along the demyelinated axons [39, 40]. We therefore assume that the capacity for myelination augments in accordance with the proportion of demyelinated axons (at least in the first 10–20 years of the disease) [18, 24]. Also, since myelogenesis primarily occurs via elongation, we assume that *M* grows in a sub-linear manner, with a coefficient *q*<1. This leads to a decrease in the capacity of oligodendrocytes to remyelinate axons over time [41–43]. The significance and the range of each term is given in Table 2 and in our simulations below, we assume that all axons are initially myelinated (*A*_*d*_ = 0, *A*_*m*_ = 1) and that the capacity of myelination is equal to the baseline healthy state (*M* = 1).

**Table 2:** Model parameters.

	Description	Unit	range
k_m_	remyelination rate (# myelin sheets per day)	1/day	[0–100]
k_md_	rate of myelinated-axonal loss per day in acute plaques (due to inflammatory infiltrates)	1/day	[0–0.01]
k_d_	rate of demyelinated-axonal loss per day in chronic plaques (due to degenerative processes)	1/day	[0–1]
*q*	cooperativity coefficient of myelin sheet production by oligodendrocytes	-	[0–1]
δ	myelination capacity growth rate	1/day	[0–1]

### Modeling autoimmune attacks

**Fig 7:**
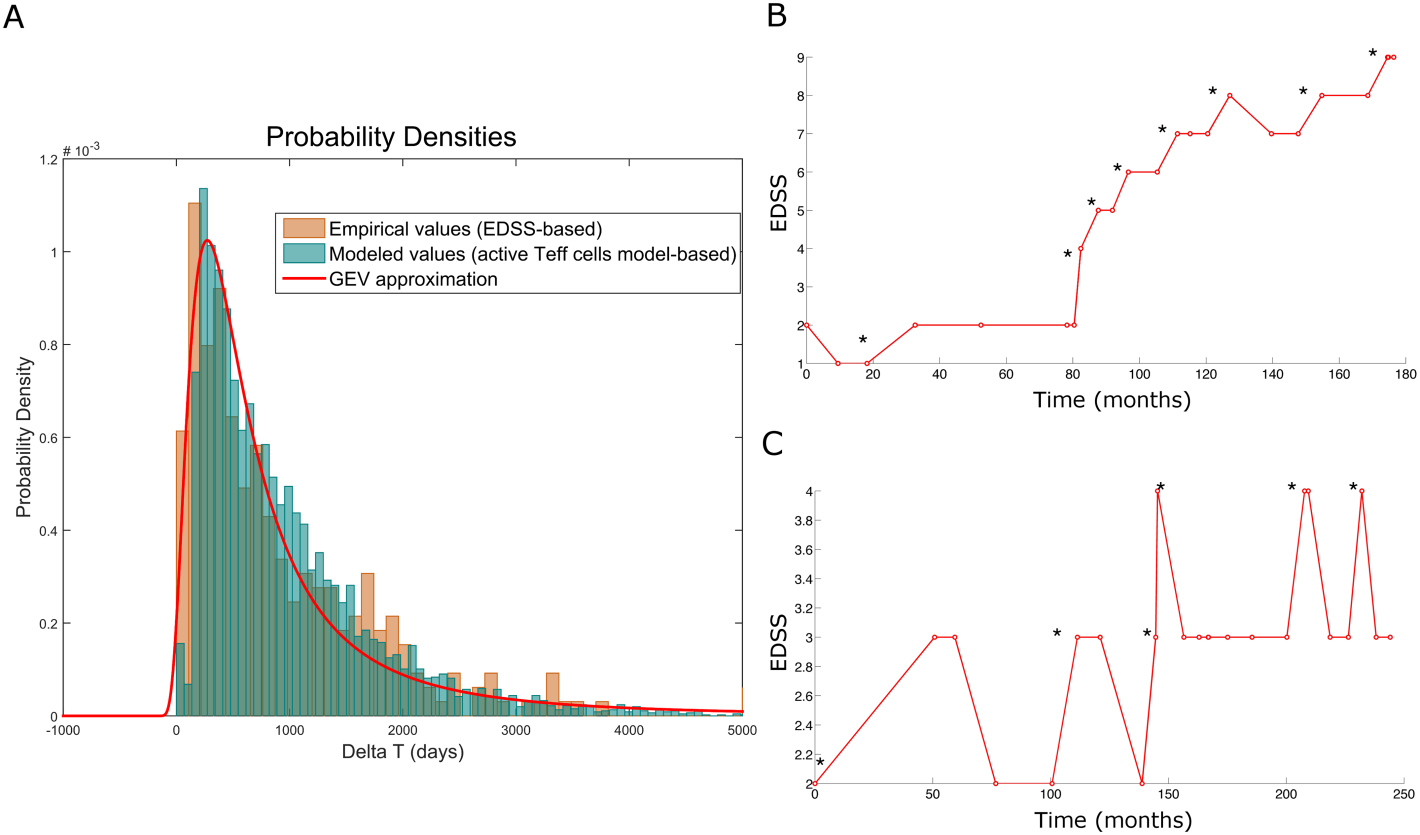
Analysis of the distribution of the EDSS time series. A) Distribution of the time intervals between the clinical relapses. The distribution is derived from the experimental data of the EDSS time series (orange), simulations from the ODE model of T cell cross-regulation [4] (light blue) and the results from the GEV distribution model (red line). B-C) Examples of the analysis of the EDSS time-series in patients with MS. Incremental changes in EDSS over time in the experimental series for (B) PPMS and (C) SPMS. The onset of ΔEDSS is marked with an asterisk.

In our model, autoimmune attack is represented by the time-dependent parameter λ(t). We modeled the inflammatory attack in terms of timing in order to capture the dynamics of the damage but for simplicity, not in terms of severity or mechanisms. In addition, we did not distinguish between different types of inflammatory damage, although the dynamics of relapsing inflammation and chronic inflammation was modeled based on the parameters. In order to define the ΔEDSS distribution, we used the timing between clinical relapses (ΔT) from the discovery cohort. Similar shapes of cluster-based ΔT distributions were obtained, with slightly different cluster-specific characteristics, allowing a further estimation of the parameters. We found that the GEV distribution of the changes in EDSS approached the simulated values, lying in the 97^th^ percentile in terms of intensity and the ΔT distributions obtained from the ODE model of T cell cross regulation in MS [4, 23] (Fig 7). Our model does not contemplate the feedback provided by CNS antigens from the damaged brain being presented again in cervical lymph nodes, mainly because such processes have still not been proven in MS patients and there is little quantitative data to model such processes [10]. We compared the distribution of the inflammatory events from the ODE model of MS autoimmunity with the ΔEDSS distribution, and both followed a similar distribution (Fig 7). For this reason and again for simplicity, we used the GEV distribution of ΔEDSS as the source of λ(t).

### Linking the model of dynamic CNS damage to clinical outcomes

In order to link the biological processes in MS that damage the CNS with the clinical phenotype, we used an intermediate scale, measuring BV by MRI as a surrogate of CNS tissue damage (which mainly reflects demyelination and axon loss but that can be confounded by inflammation and edema or pseudoatrophy) [44]. Therefore, we aimed to relate the changes in BV with those in the disability scale (*EDSS(t)*). To this end, we made use of the fact that most regions of the CNS do not produce symptoms after damage (*non-eloquent regions* or brain volume resilient *V*_*ri*_*(t)* in our notation), whereas other regions always induce clinical symptoms after being damaged (*eloquent regions* or brain volume eloquent *V*_*ei*_*(t)*)) [45].

Both eloquent and resilient regions of the brain are affected by MS at similar rates (the lesion distribution in the CNS has some areas of preference for MS, although for simplicity we assumed here that all regions have the same probability) [26, 46]. In order to compare the data obtained from different patients, we assumed that the individual brain volume *BV*_*i*_*(t)* is linearly dependent on age and disease duration, with the individual linear coefficient *k*_*bi*_ of the normalized BV *V*_*s*_*(t)* and with a constant individual parameter *V*_*di*_ (minimal BV that remains after maximum disability is achieved: EDSS = 10, patient death).

$$BV_{i}\left(t\right)=k_{bi}V_{s}\left(t\right)+\;V_{di}$$ (5)

We represent the two fractions of normalized BV *V*_*s*_*(t)* occupied by myelinated and demyelinated axons as *A*_*m*_*(t)* and *A*_*d*_*(t)*, respectively, and we denote the myelination capacity (with respect to the normalized -healthy- brain) as *M*.

$$V_{s}\left(t\right)=\;A_{m}\left(t\right)+\;A_{d}\left(t\right)$$ (6)

### Clinical readouts of the model

We linked the model’s variables to both the time-dependent individual (*i*) disability *EDSS(t)* and to the MRI-derived BV of the individual *BV*_*i*_*(t)*. We consider that clinical disability is the result of a combination of both axon degeneration *A*_*d*_*(t)* and demyelination *A*_*m*_*(t)*. Given that the EDSS assumes a non-cumulative effect of axon demyelination and degeneration, it can be represented more adequately using the maximum rather than the sum of the two components.

$$EDSS_{i}\left(t\right)=k_{ei}EDSS_{s}\left(t\right)=\;k_{ei}max\left(A_{d}\left(t\right),D\left(t\right)\right)$$ (7)

Where *k*_*ei*_ is a scaling factor that transforms the simulated *EDSS*_*s*_ derived from the volume fractions *A*_*d*_*(t)* and *D(t)* (range from 0 to 1) normalized to the observed EDSS (range: 0–10).

In contrast to *EDSS(t)*, individual brain volume *BV*_*i*_*(t)* is linearly dependent on the purely additive measure of functional axons, both myelinated *A*_*m*_*(t)* and demyelinated *A*_*d*_*(t)*, *V*_*s*_*(t)*. Thus, for each individual

$$BV_{i}\left(t\right)=k_{bi}V_{s}\left(t\right)+\;V_{di}=k_{bi}\left(A_{m}\left(t\right)+A_{d}\left(t\right)\right)+V_{di}$$ (8)

where *k*_*bi*_ is a patient-specific proportionality constant and *V*_*di*_ is the patient specific minimal BV.

### Assumptions and boundary conditions

In order to identify all the coefficients needed to connect the model to the experimental readouts, we estimated boundary conditions for the variables at specific time points (S4 Table) on the basis of biological considerations and the conservation law.

$$A_{m}\left(t\right)+A_{d}\left(t\right)+D\left(t\right)=V_{s}\left(t\right)+D\left(t\right)=constant$$ (9)

Based on the equations presented in the table, we see the linear dependence between the *BV*_*i*_*(t*_*A*_*)* and *EDSS(t*_*A*_*)* for the time points outside of the relapses, such as

$$BV_{i}\left(t_{A}\right)=B_{i}EDSS_{i}\left(t_{A}\right)+C_{i}$$ (10)

where

$$B_{i}=-k_{bi}/k_{ei}$$ (11)

$$C_{i}=k_{bi}A_{mi}\left(0\right)+V_{di}$$ (12)

And

$$V_{di}=C_{i}+k_{ei}B_{i}A_{mi}\left(0\right)$$ (13)

Taking into account that

$$EDSS_{i}\left(t_{10}\right)=k_{ei}A_{mi}\left(0\right)=EDSS_{max}$$ (14)

for each individual patient or group of patients having both BV and EDSS measurements, the constants and coefficients necessary for the model predictions could be defined from the estimation of *C*_*i*_ and *B*_*i*_ as follows:

$$k_{ei}=EDSS_{max}/A_{mi}\left(0\right)$$ (15)

$$k_{bi}=-k_{ei}B_{i}=-EDSS_{max}B_{i}/A_{mi}\left(0\right)$$ (16)

And, for each individual:

$$V_{di}=C_{i}+EDSS_{max}B_{i}$$ (17)

$$BV_{i}\left(t\right)=-k_{ei}B_{i}V_{s}\left(t\right)+\;V_{di}=-\frac{EDSS_{max}B_{i}V_{s}\left(t\right)}{A_{mi}\left(0\right)}+C_{i}+EDSS_{max}B_{i}$$ (18)

$$EDSS_{i}\left(t\right)=\;EDSS_{max}EDSS_{s}\left(t\right)/A_{mi}\left(0\right)$$ (19)

And,

$$V_{s}\left(t\right)=\;\frac{A_{mi}\left(0\right)}{EDSS_{max}B_{i}}\left(C_{i}+EDSS_{max}B_{i}-BV_{i}\left(t\right)\right)$$ (20)

$$EDSS_{s}\left(t\right)=EDSS_{i}\left(t\right)\frac{A_{mi}\left(0\right)}{EDSS_{max}}$$ (21)

Where *C*_*i*_ and *B*_*i*_ are patient-specific linear coefficients, identified based on the dependence between EDSS and BV measurements. *EDSS*_*s*_*(t)* and *V*_*s*_*(t)* are normalized simulated values, and *A*_*mi*_*(0)* is 1 for young people under 20 or dependent on the age as 1-(age of onset-20)*0.01, bearing in mind that the decline in the volume of myelinated fibers is about 1% per year [39, 40].

### Parameter estimation

**Fig 8:**
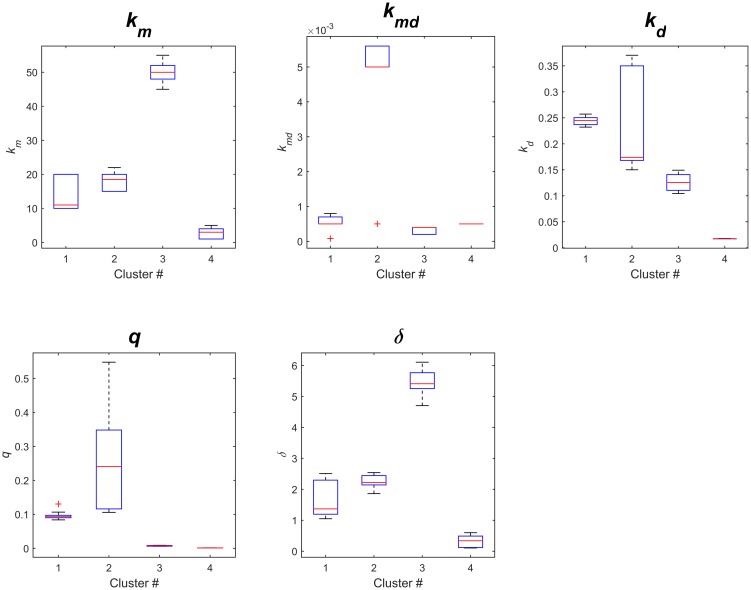
The distribution of the model’s parameters calculated for the four clusters. Each boxplot shows the distribution of each parameter in the model for each of the clusters: *km*, *kmd*, *kd*, *q*, and *δ*.

Considering the lack of quantitative data available to estimate the parameters, we approached their estimation by first obtaining parameters from the literature (S1 Table), thereafter establishing a parameter search space by setting the largest possible intervals for each parameter using the experimental EDSS time-series to restrict the parameters in the model. As such, the parameters are not assumed but rather, they are the result of fitting to the experimental dataset within biological ranges (S5 Table). The model proposed encompasses the expected linear negative correlation of the two MS read-outs, the EDSS and BV (S3 Fig). The characteristic coefficients *B*_*i*_ and *C*_*i*_ that display a linear dependence could be estimated for each patient: *V*_*e*_*(t) = C*_*i*_ *+ B*_*i*_ *· EDSS(t)*. As such, the clinical outcomes (BV and EDSS) and the simulated data can be connected at the level of the individual patient through age-based adjustments for the initial condition *A*_*mi*_(t = 0). However, as BV measurements were not available for the discovery cohort, we fitted the model to all disability scores (EDSS) for each specific cluster and based on the average initial condition *A*_*mi*_(*t* = 0) equal to 0.9. Therefore, the time-dependent parameter *λ*(t) was tuned according to the cluster-specific statistics of ΔT described above for each of the clusters. We obtained 100 random samples from the ΔT and ΔEDSS distributions of all the patients in each cluster, and we assumed that the inflammatory infiltrates decreased sharply and produced permanent CNS damage after each attack [3]. Without any loss of generality, we randomly sampled from a normal distribution with a mean of 0 to simulate the baseline diversity. The variances of the normal distributions were drawn from an inverse chi-squared distribution and the smoothed splines were then fitted to all the points sampled. Next, we used genetic algorithms to minimize the squared error between the model’s predicted values and the experimental data (EDSS). This was achieved by dividing the dataset of the discovery cohort into a training set composed of half of the EDSS data points and a test set with the other half. The data fitting provided different sets of parameters that fit the data equally well (Fig 8 and S5 Table)

Depending on the performance of the optimization algorithm (fourth order Runge–Kutta method) to search the minima, the intervals were retuned to get the best possible value for the parameter sets. Each optimization cycle runs up to 500 generations with a population size of 100 and a new population is produced for every generation using adaptive mutation function. We collectively fitted the model's parameters to a maximum 120 data points. To check the possibility that our algorithm is getting stuck in the minimum objective function (the sum of squared errors for the model), we initiated the algorithm in different parts of the objective function. Objective functions are equations to be optimized given certain constraints and with variables that need to be minimized or maximized using non-linear programming techniques. To deal with parameter uncertainty, we used an ensemble of parameter sets statistically drawn from all the sets and that were consistent with the available data. In addition, we performed a sensitivity analysis to investigate how parameter variation influenced the dynamic behavior of the model.

### Sensitivity analysis

In order to estimate the values of the model’s parameters, we used a sensitivity analysis to investigate the effects of the uncertainties in parameters on the behavior of the model and to rank the parameters in function of their effect on the output variables [47, 48]. We used the extended version of a Fourier amplitude sensitivity test (eFAST) to quantify the relative importance of the input factors. The eFAST test allows the full effect of a parameter to be quantified and the significance of the sensitivity indices to be determined, calculated by comparing with dummy parameters. Indices with very small ranges were considered as zero. The mean values of the parameters in the ODE model are given in Table 2. We assume that each parameter satisfies a uniform distribution with a coefficient of variation (CV) of 100%. The model was evaluated for each input sample to produce a set of 1000 model outputs. The median result of the eFAST test, and the full order of the sensitivity indices for brain atrophy and EDSS with respect to the model’s parameters are shown in Fig 4A.

## Supporting information

S1 File Raw data of the discovery cohort (retrospective longitudinal MS cohort). (XLSX)

S2 File Raw data of the validation cohort (prospective MS cohort). (XLSX)

S1 Table Parameter estimation from the literature. (DOCX)

S2 Table The pairwise Wilcoxon test comparing the model’s parameters between MS subtypes. (DOCX)

S3 Table Subtype specific parameters of the model. (DOCX)

S4 Table Boundary conditions for the model’s variables and readouts. (DOCX)

S5 Table Parameters of the model for each cluster. (DOCX)

S1 Fig Analysis of the cluster to calculate the top 10 parameters. We calculated the number of patients from the discovery cohort in each cluster. Based on these ratios, we computed the linear combinations of the cluster-specific parameter sets (the 10 combinations of the parameter’s values with the 10 lowest objective function values) to define the MS subtype-specific parameter sets.
(DOCX)

S2 Fig Probability of reaching EDSS ≥ 4 and EDSS ≥ 6. A) The probability of reaching an EDSS value ≥ 4 was calculated using the experimental data from the 4 clusters (red—cluster 1, green—cluster 2, blue—cluster 3, black—cluster 4). Cluster-wise simulated data (light brown lines, each line corresponds to one stochastic inflammatory input *λ*(*t*) and the probabilities are counted for the top 10 sets of parameters) and the corresponding experimental datasets (red, green, blue and black). B) The probability of reaching an EDSS value ≥ 6 was calculated using the experimental values for the 4 clusters (red—cluster 1, green—cluster 2, blue—cluster 3, black—cluster 4). The resulting curve for the discovery cohort was approximated by the Hill equation (x^b/(x^b+a)) with different coefficients for the distinct clusters. The inflection point of the Hill curve was the estimated time-point that falls exactly in the middle of the minimum and maximum EDSS values, and it was equal to 119, 25, 338 and 189 months for Cluster 1, 2, 3 and 4, respectively. This time-point could be used as a characteristic value of EDSS dynamics.
(DOCX)

S3 Fig Correlation between brain volume and EDSS. A) Brain volume versus EDSS scores in the validation cohort, where each color represents a cluster allocation. Each line corresponds to a prospective assessment of a given patient. B) Correlation coefficients *BV* ~ *EDSS* calculated for each of the individuals in the validation cohort with 3 and more data points. C) Distribution of the correlation coefficients *BV(EDSS)* using a bootstrap procedure (all patients). The two individual cases where a positive correlation was observed can be explained by relapse measurement or errors in EDSS evaluation.
(DOCX)

S4 Fig Analysis of the distribution of the EDSS time-series. A frequency distribution of the population behavior for the median time between ΔEDSS fitted by the GEV distribution (blue line). The shape of the cumulative distribution shows the goodness of the model compared to other statistical models, such as logistic, exponential or t-location scale models.
(DOCX)
