# Supplementary material for: Dynamics and heterogeneity of brain damage in multiple sclerosis
Source: PLoS Comput Biol. 2017 Oct 26;13(10):e1005757. doi: 10.1371/journal.pcbi.1005757 (PMC5657613; doi:10.1371/journal.pcbi.1005757)
Supplement: S1 Table — (DOCX) [file pcbi.1005757.s003.docx]

**S1 Table. Parameter estimation from the literature**

| **Parameter** | **Quantitative biology information** | **ref** |
| --- | --- | --- |
| *K_m_* | - Myelinating oligodendrocytes produce as much as 5–50 × 10^3^ μm^2^ of membrane each day. - An oligodendrocyte can myelinate up to 100 axons - 10 myelin sheets are produced in 10 days in vitro during LPC remyelination. - Range of the parameter: [0 - 100] (unit: 1/day). For a given unit of CNS volume number of myelin sheaths are up to 100 | [1-4]  [5] |
| *K_md_* | - The average number of transected axons (terminal axonal ovoids) per mm^3^ of tissue is 11,236 in active lesions, 3,138 in the demyelinating borders of chronic active lesions, and 875 in their inactive centers. - In acute plaques, the numbers of transected axons (APP+ elements) counted in areas of 0.01 mm^2^ were 8 (5-13), 7 (3-17) in chronic active lesions and 0 in chronic active plaques. Therefore, in 1 mm^3^ on average 0.007- 0.008 transected axons in either acute or chronic active lesions - Acute axonal loss in EAE: progressive, bilaterally symmetrical loss of dorsal corticospinal axons in the lumbar spinal cords of MOG peptide EAE mice was 56% and 26% relative to the controls on days 35 and 101, respectively, Number of axons per field (x100): 2,884 axons at baseline; 1,668 axons on day 35; and 647 axons on day 101. This gives a myelinated-axonal loss per day in the early phase (acute plaques) of 2,884-1,668/35=2.83 axons per day. The loss of demyelinated-axons per day in the chronic phase (chronic phase) was 1,668-647/66= 1.65 axons per day.   Note: We must take in consideration that the late and chronic phase of EAE is more representative of chronic active plaques than chronic inactive plaques where neurodegeneration takes place. For this reason, this ratio must be smaller.   - Range of the parameter: [0 - 0.01] (unit: 1/day). Acute axonal loss in acute and chronic active plaques are up to 0.01 per mm^3^ | [6, 7]  [8]  [9] |
| *K_d_* | - There is a reduction in axon number in the range of 60–70% compared to the normal spinal cord, with a range 20-90% depending on each case. - Axonal density (number of axons per unit area) decreases by 60% (33 to 77%). - There is an average axon reduction of 64% in demyelinated lesions of the brain, in contrast to 59% in remyelinated lesions. An average axon loss of 82% was observed in demyelinated lesions of the spinal cord. - This finding is closely recapitulated in the chronic EAE model, in which mice with permanent paralysis exhibit 59% and 43% axonal loss in the cervical and lumbar spinal cord, respectively. - Range of the parameter: [0 - 1] (unit: 1/day). This parameter ranges from full preservance of axons (1) to completely loss of all axons (0) | [6, 10, 11]  [10, 12]  [11]  [13] |
| *q* | - In the CNS, one oligodendrocyte can produce up to 100 myelin segments on multiple axons. - Range of the parameter: [0 - 1] (adimensional). This parameter ranges from full myelination (1) to complete demyelination (0) | [1-3] |
| *M_total_* | - The surface area of myelin per adult rat oligodendrocyte is 1-20 × 10^5^ µm^2^ - In Rhesus monkey, the Myelin Volume Fraction (MVF) is 0.2725 the Axonal/fiber volume fraction (FVF) is 0.55125, the Aggregate g-ratio is 0.715 and the Mean g-ratio is 0.64625. - In the monkey corpus callosum (CC), unmyelinated axons account for about 30% of all axons in the genu of the corpus callosum (CC) and less than 10% elsewhere in the CC, with a small caliber (<0.1 µm). - Range for δ: [0 - 1], units 1/day. The parameter ranges from full myelination (1) to complete demyelination (0) | [3]  [14]  [15] |

**References**

1. Snaidero N, Simons M. Myelination at a glance. Journal of cell science. 2014;127(Pt 14):2999-3004. doi: 10.1242/jcs.151043. PubMed PMID: 25024457.

2. Yeung MS, Zdunek S, Bergmann O, Bernard S, Salehpour M, Alkass K, et al. Dynamics of oligodendrocyte generation and myelination in the human brain. Cell. 2014;159(4):766-74. doi: 10.1016/j.cell.2014.10.011. PubMed PMID: 25417154.

3. Pfeiffer SE, Warrington AE, Bansal R. The oligodendrocyte and its many cellular processes. Trends Cell Biol. 1993;3(6):191-7. PubMed PMID: 14731493.

4. Miron VE, Kuhlmann T, Antel JP. Cells of the oligodendroglial lineage, myelination, and remyelination. Biochim Biophys Acta. 2011;1812(2):184-93. doi: 10.1016/j.bbadis.2010.09.010. PubMed PMID: 20887785.

5. Vereyken EJ, Fluitsma DM, Bolijn MJ, Dijkstra CD, Teunissen CE. An in vitro model for de- and remyelination using lysophosphatidyl choline in rodent whole brain spheroid cultures. Glia. 2009;57(12):1326-40. doi: 10.1002/glia.20852. PubMed PMID: 19191324.

6. Kutzelnigg A, Lassmann H. Pathology of multiple sclerosis and related inflammatory demyelinating diseases. Handbook of clinical neurology. 2014;122:15-58. doi: 10.1016/B978-0-444-52001-2.00002-9. PubMed PMID: 24507512.

7. Trapp BD, Peterson J, Ransohoff RM, Rudick R, Mork S, Bo L. Axonal transection in the lesions of multiple sclerosis. N Engl J Med. 1998;338(5):278-85.

8. Ferguson B, Matyszak MK, Esiri MM, Perry VH. Axonal damage in acute multiple sclerosis lesions. Brain. 1997;120 ( Pt 3):393-9. PubMed PMID: 9126051.

9. Soulika AM, Lee E, McCauley E, Miers L, Bannerman P, Pleasure D. Initiation and progression of axonopathy in experimental autoimmune encephalomyelitis. J Neurosci. 2009;29(47):14965-79. Epub 2009/11/27. doi: 29/47/14965 [pii]

10.1523/JNEUROSCI.3794-09.2009. PubMed PMID: 19940192; PubMed Central PMCID: PMC2990681.

10. Bjartmar C, Kidd G, Mork S, Rudick R, Trapp BD. Neurological disability correlates with spinal cord axonal loss and reduced N-acetyl aspartate in chronic multiple sclerosis patients. Ann Neurol. 2000;48(6):893-901.

11. Mews I, Bergmann M, Bunkowski S, Gullotta F, Bruck W. Oligodendrocyte and axon pathology in clinically silent multiple sclerosis lesions. Mult Scler. 1998;4(2):55-62. PubMed PMID: 9599334.

12. Popescu BF, Lucchinetti CF. Pathology of demyelinating diseases. Annu Rev Pathol. 2012;7:185-217. doi: 10.1146/annurev-pathol-011811-132443. PubMed PMID: 22313379.

13. Wujek JR, Bjartmar C, Richer E, Ransohoff RM, Yu M, Tuohy VK, et al. Axon loss in the spinal cord determines permanent neurological disability in an animal model of multiple sclerosis. J Neuropathol Exp Neurol. 2002;61(1):23-32. PubMed PMID: 11829341.

14. Stikov N, Campbell JS, Stroh T, Lavelee M, Frey S, Novek J, et al. Quantitative analysis of the myelin g-ratio from electron microscopy images of the macaque corpus callosum. Data Brief. 2015;4:368-73. doi: 10.1016/j.dib.2015.05.019. PubMed PMID: 26217818; PubMed Central PMCID: PMCPMC4510539.

15. LaMantia AS, Rakic P. Axon overproduction and elimination in the anterior commissure of the developing rhesus monkey. J Comp Neurol. 1994;340(3):328-36. doi: 10.1002/cne.903400304. PubMed PMID: 8188854.
