# Supplementary material for: Dynamics and heterogeneity of brain damage in multiple sclerosis
Source: PLoS Comput Biol. 2017 Oct 26;13(10):e1005757. doi: 10.1371/journal.pcbi.1005757 (PMC5657613; doi:10.1371/journal.pcbi.1005757)
Supplement: S2 Table — (DOCX) [file pcbi.1005757.s004.docx]

**S2 Table.** **P values of the pairwaise Wilcoxon test of the comparison of model parameters between MS subtypes**

| **p-values**  **(Wilcoxon test)** | ***Km*** | ***Kmd*** | ***Kd*** | ***q*** | ***δ*** |
| --- | --- | --- | --- | --- | --- |
| RRMS - SPMS | 0.0002 | 0.0001 | 0.0002 | 0.06 | 0.001 |
| SPMS - PPMS | 0.009 | 0.001 | 0.05 | 0.06 | 0.02 |
| RRMS - PPMS | 0.0002 | 8E-05 | 0.0002 | 0.0002 | 0.0002 |
