# Supplementary material for: Dynamics and heterogeneity of brain damage in multiple sclerosis
Source: PLoS Comput Biol. 2017 Oct 26;13(10):e1005757. doi: 10.1371/journal.pcbi.1005757 (PMC5657613; doi:10.1371/journal.pcbi.1005757)
Supplement: S3 Table — (DOCX) [file pcbi.1005757.s005.docx]

**S3 Table. Subtype specific parameters of the model**

|  | *K_m_* | *δ* | *K_d_* | *K_md_* | *q* |
| --- | --- | --- | --- | --- | --- |
| RRMS median | 24.71429 | 2.740742 | 0.095745 | 0.000755 | 0.027345 |
| IQR | 2.357143 | 0.41932 | 0.0037 | 0.0001 | 0.01716 |
| SPMS median | 20.3125 | 2.422901 | 0.1445 | 0.002 | 0.1145 |
| IQR | 3.1 | 0.424787 | 0.067 | 0.001 | 0.095 |
| PPMS median | 16.8185 | 2.056792 | 0.194 | 0.004 | 0.2005 |
| IQR | 5.818 | 0.430303 | 0.132 | 0 | 0.173 |
