# Supplementary material for: Dynamics and heterogeneity of brain damage in multiple sclerosis
Source: PLoS Comput Biol. 2017 Oct 26;13(10):e1005757. doi: 10.1371/journal.pcbi.1005757 (PMC5657613; doi:10.1371/journal.pcbi.1005757)
Supplement: S4 Table — (DOCX) [file pcbi.1005757.s006.docx]

**S4 Table. Boundary conditions for model variables and readouts**

| **Time points** | ***t = t_0_*** | ***t = t_10_*** | ***t = t_A_*** |
| --- | --- | --- | --- |
| Description | Healthy state | Maximum EDSS=10 (death) | Remission (out of relapse) |
| Comments | Healthy, grown up state. All axons in *V_s_(0)* are myelinated to their individually adjusted maximum *A_mi_(0)*. Myelination capacity 1 is equal to the baseline healthy state | Death state. Almost all eloquent axons are transected. | The model assumes that axons at any time point far enough from the relapses is either normally myelinated or transected. |
| $A_{m}\left( t \right)$ | $A_{mi}\left( 0 \right)$ | 0 | $A_{m}\left( t_{A} \right)$ |
| $A_{d}(t)$ | 0 | 0 | 0 |
| $D(t)$ | 0 | $A_{mi}\left( 0 \right)$ | $D\left( t_{A} \right)$ |
| $M(t)$ | 1 | $M(t_{10})$ | $M(t_{A})$ |
| ${EDSS}_{i}(t)=$  $k_{ei}max(A_{d}(t),D(t))$ | ${EDSS}_{i}\left( t_{0} \right)=0$ | ${EDSS}_{i}\left( t_{10} \right)=$  $k_{ei}A_{mi}\left( 0 \right)={EDSS}_{max}$ | ${EDSS}_{i}\left( t_{A} \right)= k_{ei}D\left( t_{A} \right)=$  $k_{ei}{(A}_{mi}\left( 0 \right)-A_{m}\left( t_{A} \right))$ |
| ${BV}_{i}\left( t \right)=$  $k_{bi}(A_{m}(t)+A_{d}(t))+V_{di}$ | ${BV}_{i}\left( t_{0} \right)=$  $k_{bi}A_{mi}\left( 0 \right)+V_{di}$ | ${BV}_{i}\left( t_{10} \right)=V_{di}$ | ${BV}_{i}\left( t_{A} \right)=k_{bi}A_{m}\left( t_{A} \right)+V_{di}$ |
