# Supplementary material for: Dynamics and heterogeneity of brain damage in multiple sclerosis
Source: PLoS Comput Biol. 2017 Oct 26;13(10):e1005757. doi: 10.1371/journal.pcbi.1005757 (PMC5657613; doi:10.1371/journal.pcbi.1005757)
Supplement: S5 Table — (DOCX) [file pcbi.1005757.s007.docx]

**S5 Table. Parameters of the model for each cluster**

|  | *k_m_* | *k_md_* | *k_d_* | *q* | *δ* |
| --- | --- | --- | --- | --- | --- |
| Cluster 1 Median | 11 | 0.0005 | 0.24482 | 0.09322 | 1.368716 |
| IQR | 10 | 0.0002 | 0.01338 | 0.00724 | 1.101113 |
| Cluster 2 Median | 18.5 | 0.005 | 0.17395 | 0.24075 | 2.220546 |
| IQR | 5 | 0.0006 | 0.1819 | 0.2321 | 0.301587 |
| Cluster 3 Median | 50 | 0.0004 | 0.1255 | 0.0076 | 5.411369 |
| IQR | 4 | 0.0002 | 0.0303 | 0.0021 | 0.512621 |
| Cluster 4 Median | 3 | 0.0005 | 0.017 | 0.0013 | 0.339 |
| IQR | 3 | 0 | 0.0004 | 0.0002 | 0.369302 |
