# Supplementary material for: Dynamics and heterogeneity of brain damage in multiple sclerosis
Source: PLoS Comput Biol. 2017 Oct 26;13(10):e1005757. doi: 10.1371/journal.pcbi.1005757 (PMC5657613; doi:10.1371/journal.pcbi.1005757)

**S1 Figure. Analysis of cluster for calculating top 10 parameters**. We calculated number of patients for each cluster in discovery cohort. Based on these ratios, we computed linear combinations of the cluster-specific parameter sets (the 10 combinations of parameters with the 10 lowest objective function values) to define MS subtype-specific parameters sets.


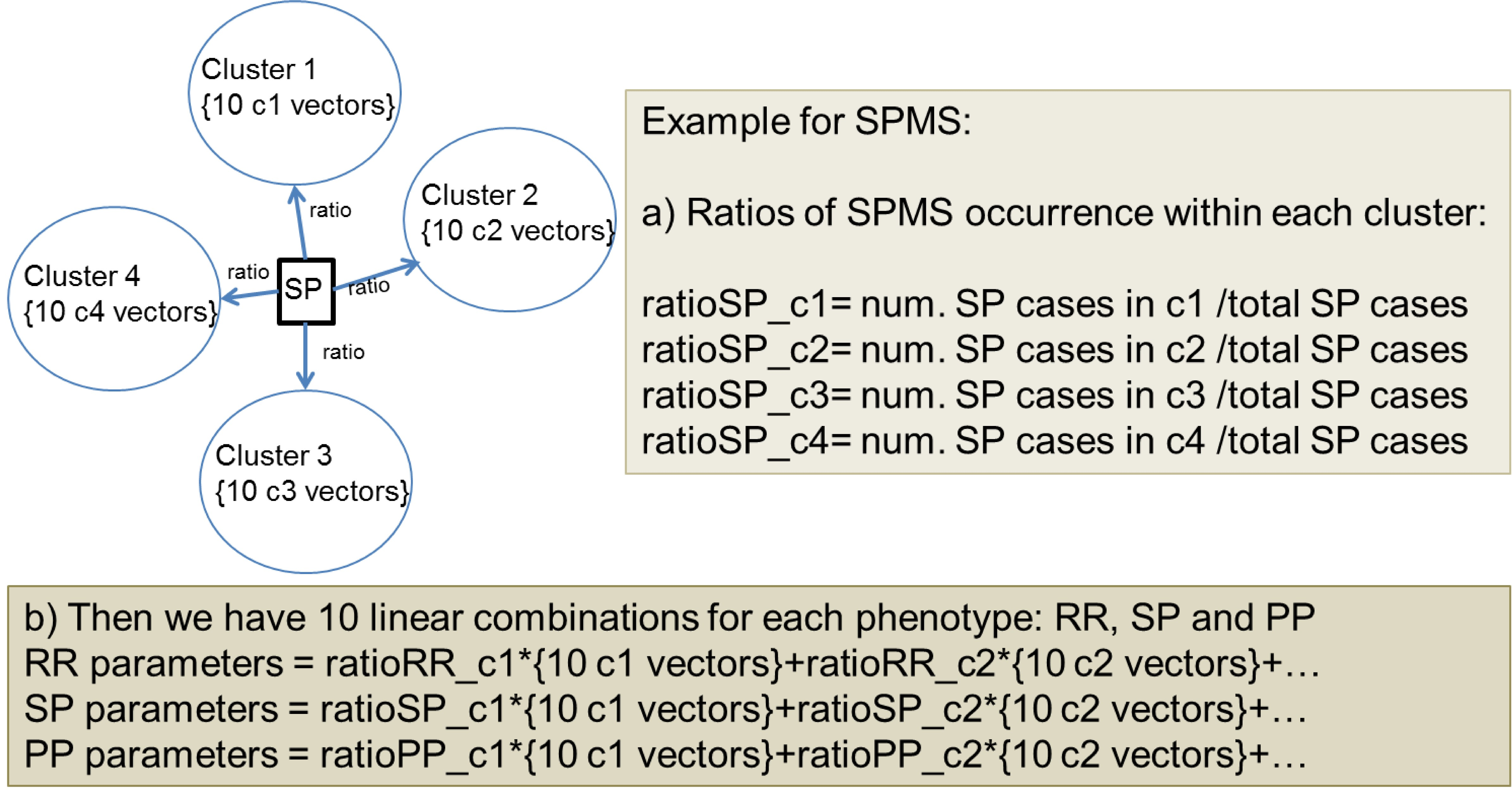

Supplement: S1 Fig — We calculated the number of patients from the discovery cohort in each cluster. Based on these ratios, we computed the linear combinations of the cluster-specific parameter sets (the 10 combinations of the parameter’s values with the 10 lowest objective function values) to define the MS subtype-specific parameter sets. (DOCX) [file pcbi.1005757.s008.docx]
