# Supplementary material for: Dynamics and heterogeneity of brain damage in multiple sclerosis
Source: PLoS Comput Biol. 2017 Oct 26;13(10):e1005757. doi: 10.1371/journal.pcbi.1005757 (PMC5657613; doi:10.1371/journal.pcbi.1005757)
Supplement: S2 Fig — A) The probability of reaching an EDSS value ≥ 4 was calculated using the experimental data from the 4 clusters (red—cluster 1, green—cluster 2, blue—cluster 3, black—cluster 4). Cluster-wise simulated data (light brown lines, each line corresponds to one stochastic inflammatory input λ(t) and the probabilities are counted for the top 10 sets of parameters) and the corresponding experimental datasets (red, green, blue and black). B) The probability of reaching an EDSS value ≥ 6 was calculated using the experimental values for the 4 clusters (red—cluster 1, green—cluster 2, blue—cluster 3, black—cluster 4). The resulting curve for the discovery cohort was approximated by the Hill equation (x^b/(x^b+a)) with different coefficients for the distinct clusters. The inflection point of the Hill curve was the estimated time-point that falls exactly in the middle of the minimum and maximum EDSS values, and it was equal to 119, 25, 338 and 189 months for Cluster 1, 2, 3 and 4, respectively. This time-point could be used as a characteristic value of EDSS dynamics. (DOCX) [file pcbi.1005757.s009.docx]

**S2 Figure. Probability of reaching EDSS > 4 and EDSS > 6.** A) Probability of reaching an EDSS value > 4 calculated using experimental data for the 4 clusters (red - cluster 1, green - cluster 2, blue - cluster 3, black - cluster 4). Cluster-wise simulated data (light brown lines, each line corresponds to one stochastic inflammatory input $\lambda(t)$ and the probabilities are counted for the top 10 sets of parameters) and the corresponding experimental datasets (red, green, blue, black). B) Probability of reaching an EDSS value > 6 calculated using the experimental values for the 4 clusters (red - cluster 1, green - cluster 2, blue - cluster 3, black - cluster 4). The resulting curve for the discovery cohort was approximated by the Hill equation (x^b/(x^b+a)) with different coefficients for distinct clusters. The inflection point of the Hill curve was the estimated time-point that falls exactly in the middle of the minimum and maximum EDSS values, and it was equal to 119, 25, 338 and 189 months for Cluster 1, 2, 3 and 4, respectively, and it could be used as a characteristic value of the EDSS dynamics.

**
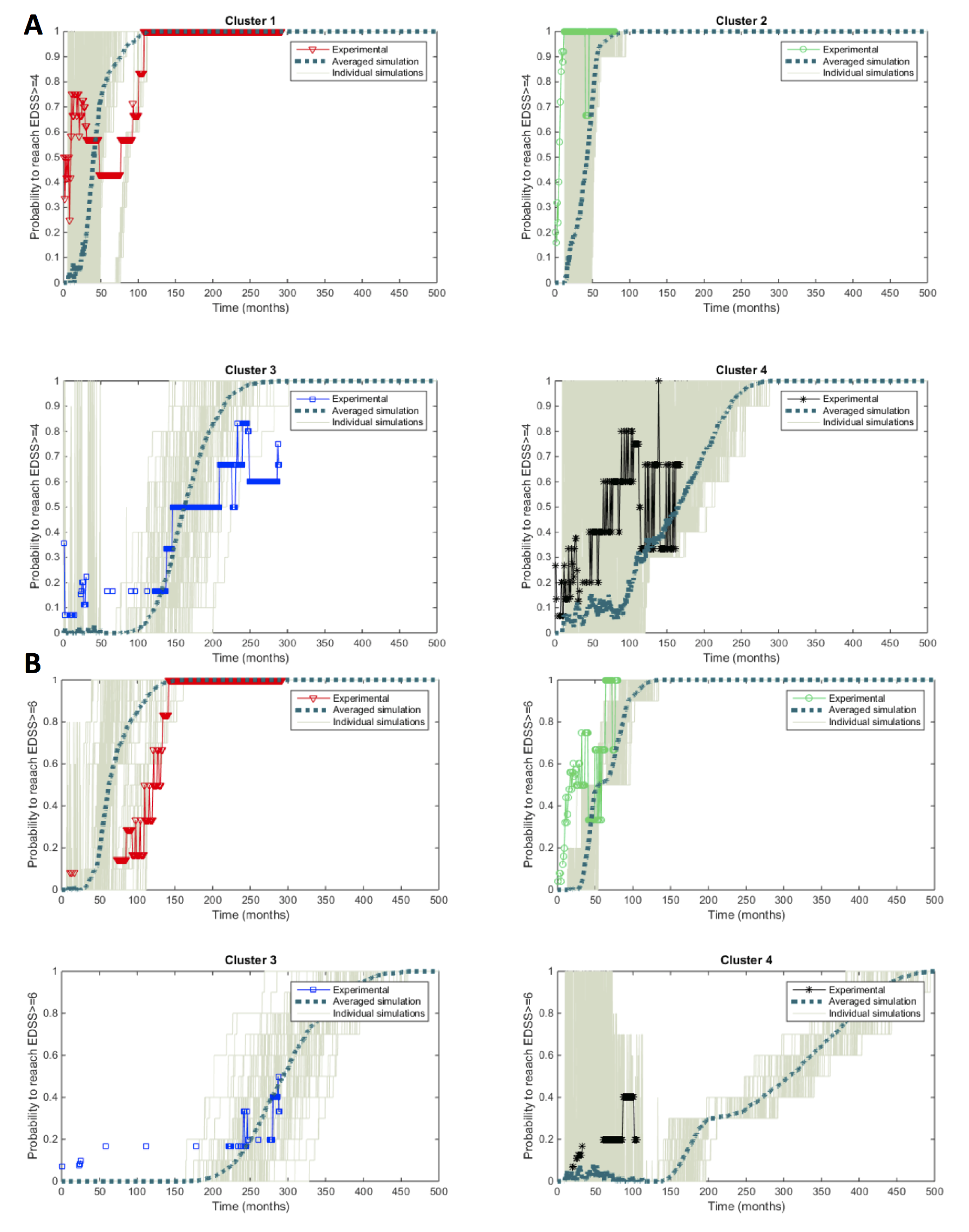
**
