# Supplementary material for: Dynamics and heterogeneity of brain damage in multiple sclerosis
Source: PLoS Comput Biol. 2017 Oct 26;13(10):e1005757. doi: 10.1371/journal.pcbi.1005757 (PMC5657613; doi:10.1371/journal.pcbi.1005757)

**S3 Figure. Correlation between brain volume and EDSS.** A) Brain volume versus EDSS scores in the validation cohort, where each color represents a cluster allocation. Each line corresponds to a prospective assessment of a given patient. B) Correlation coefficients *Brain Volume* ~ *EDSS* calculated for each of the individuals in the validation cohort with 3 and more data-points. C) Distribution of correlation coefficients *BV(EDSS)* using bootstrap procedure (all patients). The two individual cases where a positive correlation was observed can be explained by relapse measurement or errors in EDSS evaluation.


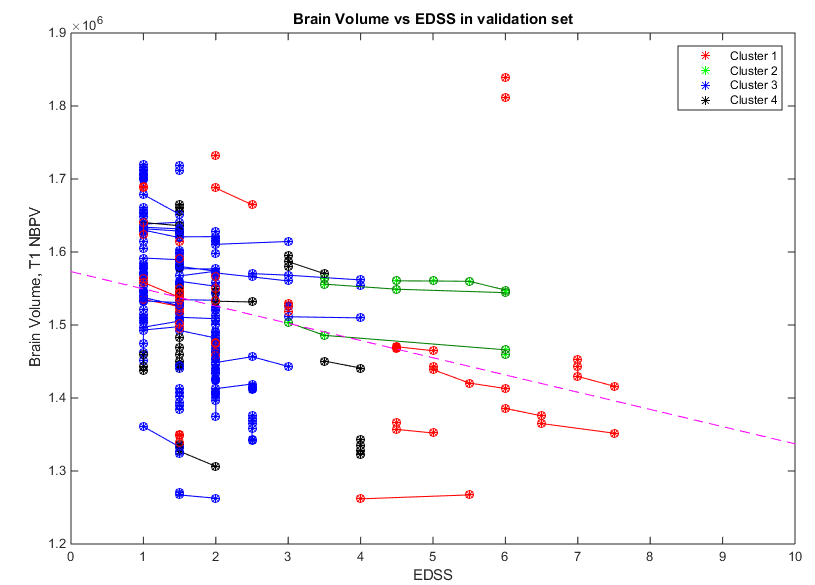

Supplement: S3 Fig — A) Brain volume versus EDSS scores in the validation cohort, where each color represents a cluster allocation. Each line corresponds to a prospective assessment of a given patient. B) Correlation coefficients BV ~ EDSS calculated for each of the individuals in the validation cohort with 3 and more data points. C) Distribution of the correlation coefficients BV(EDSS) using a bootstrap procedure (all patients). The two individual cases where a positive correlation was observed can be explained by relapse measurement or errors in EDSS evaluation. (DOCX) [file pcbi.1005757.s010.docx]
