# Supplementary material for: Dynamics and heterogeneity of brain damage in multiple sclerosis
Source: PLoS Comput Biol. 2017 Oct 26;13(10):e1005757. doi: 10.1371/journal.pcbi.1005757 (PMC5657613; doi:10.1371/journal.pcbi.1005757)

**S4 Figure. Analysis of the distribution of the EDSS time-series.** Frequency distribution of the population behavior for medians of times between ∆EDSS fitted by the GEV distribution (blue line). The shape of the cumulative distribution shows the goodness of the model compared to other statistical models, such as logistic, exponential or t-location scale models.


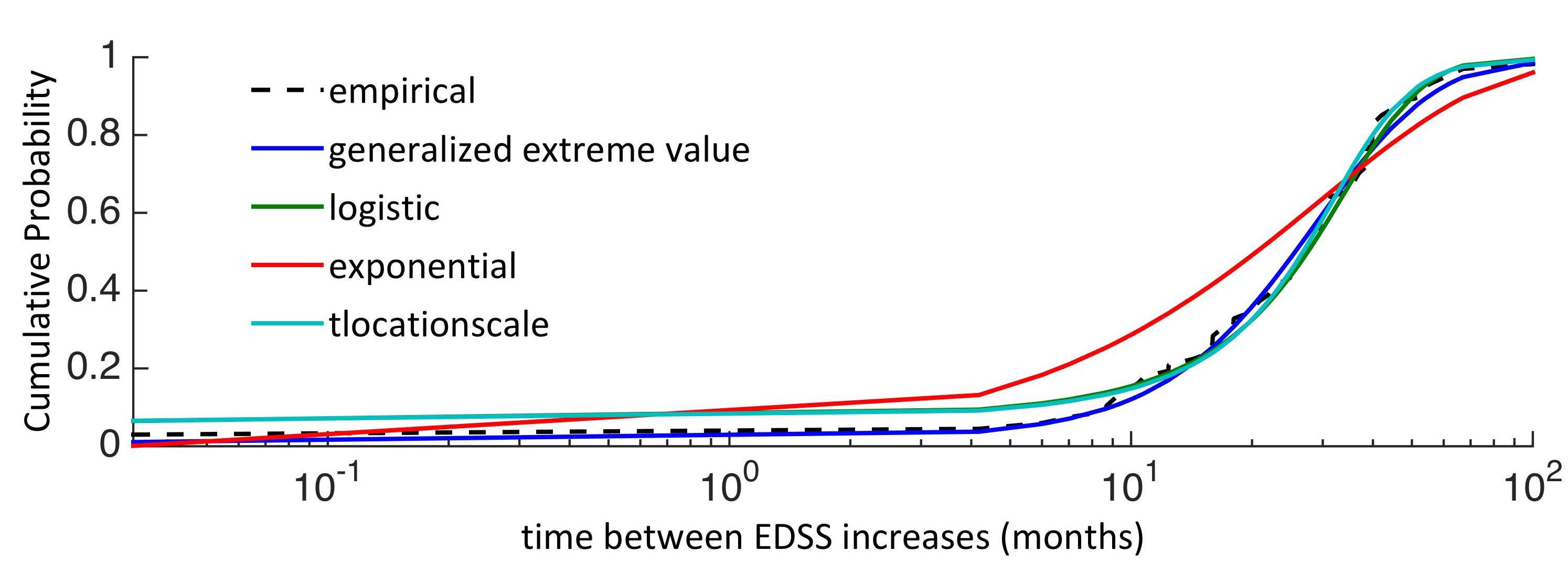

Supplement: S4 Fig — A frequency distribution of the population behavior for the median time between ΔEDSS fitted by the GEV distribution (blue line). The shape of the cumulative distribution shows the goodness of the model compared to other statistical models, such as logistic, exponential or t-location scale models. (DOCX) [file pcbi.1005757.s011.docx]
